# The economic and social burden of pediatric cerebral palsy in Spain: a cost-of-illness study

**DOI:** 10.3389/fpubh.2025.1589114

**Published:** 2025-07-23

**Authors:** Diana Marcela Nova-Díaz, Paloma Arana-Rivera, Eduardo Sánchez-Iriso, Sergio Aguilera-Albesa

**Affiliations:** ^1^Department of Economics, Public University of Navarra, Paediatric Neurology Research Group, Navarrabiomed, Pamplona, Spain; ^2^University Hospital of Navarra, Paediatric Neurology Research Group, Navarrabiomed, Pamplona, Spain; ^3^Department of Economics, Public University of Navarra, Pamplona, Spain

**Keywords:** cost of illness, economic burden, cerebral palsy, social costs, caregiver burden, healthcare costs

## Abstract

**Background:**

Cerebral palsy (CP) is the leading cause of motor disability in children and a lifelong condition with no cure, imposing a significant economic burden on families and healthcare systems. However, the economic impact of pediatric CP remains underexplored in Spain, hindering the development of cost-effective policies. Cost-of-illness (COI) studies are essential to quantify disease burden and guide resource allocation. This study aims to classify and estimate the economic and social costs of pediatric CP in Spain from a societal perspective, considering healthcare, government, and family burdens. Additionally, it evaluates the caregiving burden experienced by primary caregivers.

**Methods:**

A bottom–up, disease-specific COI study was conducted from a societal perspective using data from a population-based epidemiological registry of CP. Data collection included structured questionnaires and administrative records from regional healthcare and government sources, covering a 1-year period. The Zarit Burden Interview was used to assess caregiver burden. The study captures direct, indirect, and out-of-pocket costs, including productivity losses associated with caregiving.

**Results:**

The study included 148 children with CP (mean age: 9.72) and their primary caregivers (66% female, mean age: 42.97 years). Medical care costs averaged €3,801 (3.72%), while out-of-pocket expenses totalled €7,041 (6.89%), largely driven by complementary and alternative therapies used by 64% of familie**s**. Special education represented €8,932 (8.75%), whereas caregiver productivity losses were the largest component (€60,638; 59.37%). The mean annual societal cost per child was €102,135, over thirty times Spain's mean per capita healthcare expenditure. However, using a conservative assumption that valued the caregiver's time at the minimum wage, the social costs would be €70,190 per child. Children with severe motor impairment (GMFCS III–V) had nearly twice the cost of those with milder impairments (GMFCS I–II) (1.96; 95% CI: 1.92–2.01).

**Conclusions:**

The economic burden of pediatric CP is largely driven by caregiving and non-medical costs, highlighting gaps in financial and social support. These findings call for targeted policies to reduce caregiver strain and enhance funding for assistive services, improving equity in CP care. Additionally, comprehensive cost-effectiveness analyses are needed to guide resource allocation and ensure sustainable support strategies.

## 1 Introduction

Cerebral palsy (CP) is a group of permanent movement and posture disorders resulting from non-progressive abnormalities in the developing brain (1). It is the most common cause of severe physical disability in childhood and is often associated with comorbidities such as epilepsy, intellectual disability, feeding difficulties, and sensory impairments including hearing and vision loss (2). Managing CP requires comprehensive, multidisciplinary care, which places a significant burden not only on healthcare systems but also on families and society as a whole (3, 4).

In developed countries, the prevalence of CP is estimated at ~2.4 per 1,000 live births (5). While there is no cure, therapeutic interventions aim to improve the quality of life for affected children and their caregivers (6, 7). However, motor complications particularly spasticity, present in 70–91% of cases add complexity to disease management, leading to additional healthcare needs and associated costs. Spasticity can result in pain, sleep disturbances, and difficulties with daily activities, often requiring specialized therapeutic interventions that further increase the economic burden (8, 9). CP represents a significant economic and social challenge, particularly in terms of healthcare expenses, indirect costs, and caregiver burden. Understanding these costs is essential for designing targeted public policies that ensure equitable access to healthcare and social support.

The economic impact of CP has been documented mainly in countries such as Australia, Canada, China, and the United States, where Cost of Illness (COI) studies have highlighted the substantial financial burden on families and society (3, 10). These costs encompass direct medical expenses, productivity losses due to caregiving, and out-of-pocket costs for specialized equipment, therapies, transportation, and complementary treatments many of which are not fully covered by healthcare systems (11). A systematic review of the costs of CP worldwide estimated that medical costs for children with CP were 10–26 times higher than for healthy children, showing a *positive relationship* between the *severity of gross motor impairment and expenses* (10, 12, 13). The most recent and comprehensive estimate of the cost of CP was developed in Australia in 2018 and found a cost of around €90,597 per person per year from a societal perspective (14), while another reported that families spend a mean of €57,000 on early intervention programs and other necessary services (11). In Europe, a 2010 Dutch study estimated an annual cost of up to €40,265 per patient, excluding productivity loss (9), whereas in China, a 2003 study calculated an annual cost of €63,785 from a social perspective (4). Despite these data, the direct costs borne by families, which are often not covered by health systems, represent a significant financial burden, often forcing trade-offs in essential goods or activities (11, 15).

Beyond financial costs, CP imposes a substantial emotional and social burden on primary caregivers, often generating financial stress and caregiving overload (11). Parents of children with CP frequently face emotional distress, social limitations, and physical health concerns (16). Although these factors have been evaluated in other chronically ill populations, their analysis in caregivers of children with CP remains limited (13, 17). Addressing this gap is essential for comprehensive economic analyses (11). The inclusion of this dimension should form part of the rationale for introducing costly medical treatments and guiding funding policies (18).

This substantial economic and social burden has been moderately documented internationally, but data on CP in Spain remain scarce. The absence of a national registry complicates accurate prevalence estimates; however, based on European data, it is estimated that CP affects ~2–2.3 per 1,000 live births, which corresponds to a population prevalence of around 120,000 individuals in Spain (19, 20). The allocation of resources for CP care remains limited and unevenly distributed across regions, largely due to the lack of reliable data and proper identification of these patients (21, 22). Despite increasing financial pressures on healthcare systems, the costs of CP treatment in Spain have not been systematically evaluated, and comprehensive studies remain scarce across Europe. To our knowledge, no COI study has assessed the economic burden of CP in Spain from a societal perspective. This gap prevents policy makers from developing cost-effective health policies that promote equitable access to care and financial support for affected families (19, 23).

To address this gap, this study conducts an analysis of COI from a societal perspective, using a bottom–up approach in a regional setting in Spain. The study aims to: (1) classify and calculate the costs associated with CP from multiple perspectives, including those of society, government, healthcare systems, and families; and (2) assess the care burden experienced by primary caregivers of children with CP. The aim is to provide valuable data for policy makers and health planners.

## 2 Materials and methods

This study was carried out in collaboration with various entities specialized in the care of patients with CP, such as the Department of Education of Navarra through the Resource Center for Educational Equity in Navarra (CREENA), the University Hospital of Navarra (HUN) and the Association of Patients with Cerebral Palsy of Navarre (ASPACE). These institutions played a key role in the identification of patients and their data for the Cerebral Palsy Study of Navarra (EPCINA), to which this research is linked. Likewise, these entities, together with the families and health professionals of the Pediatric Neurology Unit of the HUN, collaborated in the design of the questionnaire used to measure the costs associated with CP.

### 2.1 Study design

A COI study was conducted using a bottom–up, disease-specific approach based on prevalence and empirical data, considering the perspectives of society, the healthcare system, the government, and families of children with CP (24, 25).

#### 2.1.1 Time frame and cost perspective

In a COI study based on prevalence and empirical evidence, costs are calculated over a defined time frame for all affected patients. For this study, a 1-year period was considered. The cost perspective defines the viewpoint from which the analysis is conducted, influencing the types of costs included and the conclusions that can be drawn (26). The societal perspective considered the most comprehensive, was adopted to estimate the economic impact of CP on society (24). Given that the study population consisted of children, conservatively only productivity losses for primary caregivers were included, reflecting the economic burden associated with caregiving (12, 27).

#### 2.1.2 Participant recruitment

In Navarra, a region in northern Spain with a pediatric population (0–18 years old) of 134.898 children (28), Project EPCINA calculated a prevalence of ~1.49 cases per 1,000 children, according to the 2023 population-based epidemiological registry from the HUN. Based on this prevalence, the estimated number of children with CP in the region would be around 201. However, according to the Cerebral Palsy Surveillance in Europe (SCPE) (19), an official diagnosis of CP is only confirmed at 3 years of age.

Children with CP and their families were initially identified through the HUN CP epidemiological registry, which provided access to the entire pediatric CP population in the region. Subsequently, a chain recruitment strategy was implemented in collaboration with institutions responsible for the allocation of resources to pediatric CP patients in Navarra. These institutions either sent email invitations or directly informed families who had previously agreed to receive research participation requests.

To be included in the study, children had to meet the following *inclusion criteria*: a confirmed CP diagnosis, age between 3 and 18 years, and residency in Navarra. Families were also required to provide informed consent and report on health resource utilization. Given that CP diagnosis is not definitive before the age of 3, and that this is the age at which schooling begins, children under 3 years old were excluded (19). Additional *exclusion criteria* included children with unconfirmed diagnoses or families unable to complete the required assessments.

Of the 201 identified cases, 49 children were under the age of 3 at the time of data collection and were excluded for not meeting the inclusion criteria, in accordance with SCPE clinical guidelines. In addition, 4 children had passed away before the study was completed, and their families understandably opted not to continue participation. Therefore, the final eligible population comprised 148 children aged 3–18 years with a confirmed CP diagnosis. All eligible families were successfully contacted and agreed to participate. No refusals, dropouts, or unreachable cases were recorded. A summary of the selection process is presented in Supplementary Figure S1.

As a result, the final sample comprised 148 children, representing ~100% of the estimated CP population in the region. This ensured comprehensive coverage of cases across different severity levels and functional classifications. The recruitment strategy aligns with previous COI studies in CP, which often rely on regional registries and clinical networks for participant identification (11, 14).

#### 2.1.3 Survey process

Survey data were collected through face-to-face interviews using a semi-structured questionnaire. Interviews were conducted by two experienced members of the research team, a health economist and a pediatric neurologist, both familiar with working with caregivers of children with CP and with administering the survey instrument. Caregivers were identified through a snowball recruitment process involving three collaborating institutions: The Hospital Universitario de Navarra (via its epidemiological registry), the regional branch of ASPACE, and CREENA.

Eligible participants were contacted by email and invited to take part in a voluntary interview. The survey began with an information sheet outlining the study's purpose, the research team, data protection procedures, and the estimated duration (30–45 min). Participation required explicit consent, documented by selecting the checkbox “I have read the participant information sheet.”

Each interview was conducted in a single sitting using printed booklets to record responses. The interviewer read each item aloud, offered clarifications when necessary, and filled in the responses in real time to ensure accuracy and completeness. All data were anonymised and subsequently entered into a structured Excel database. Participants were free to pause the interview, withdraw at any time, or review and modify their responses during the session. The survey was carried out between July 2023 and December 2024.

### 2.2 Data collection

The study employed a multisource data collection strategy that *combined primary data*, collected directly *from caregivers, with administrative data* retrieved *from healthcare, educational, and social service records*. Primary data were gathered through face-to-face interviews with primary caregivers using structured instruments to obtain detailed information on household-level expenditures, informal care time, and perceived caregiver burden (see Table 1, Parts 2 and 3).

**Table 1:** Categories of resource use included in questionnaire and diary.

**Categories of resource use included in questionnaire and diary**
**Questionnaire, part 1: Health care costs of the public health care system and government (One-year time period)**
General practitioner care Outpatient specialist medical care Diagnostic test Treatments directly related to CP comorbidities Hospitalization Emergency department attendances Ambulance transfers Prescribed medication Special equipment and aids for mobility (Wheelchair, splints, standing frame, etch) Conventional treatment/therapies^a^ Special or adapted education Personal budget (Disability allowance)
**Questionnaire, part 2: Out-of-pocket costs (variable time period, see text)**
Complementary and alternative treatments/therapies^b^ Special equipment and aids for mobility and communication that are not covered or not frequently provided. Special Clothing, Nutrition and Personal Care Over-the-counter medication Subscriptions to patients' associations Leisure, Respite Care and Holidays Transportation and fuel Wheelchair adaptations Purchase of vehicles and/or their adaptation Home modifications
**Questionnaire, part 3: Diary (one-month time period) INDIRECT ECONOMIC BURDEN: Productivity losses**
Household care Personal care Time to attend conventional therapies from the public health system Time to attend public health system medical appointments Time to attend occupational, physical and speech therapy paid for by the family Time to attend outpatient rehabilitation paid for by the family Time to attend alternative therapies paid for by families Activating and Supportive guidance

^**a**^Treatment that is included in standard medical practice or within the public health system to treat CP and its comorbidities

^**b**^Medical products and practices that are not part of standard medical care.

The data included in Part 1 of the questionnaire come mainly from **administrative sources** (e.g., medical records, hospital accounting, and records from educational and social services), particularly for categories such as medical care, conventional therapies, adapted education, disability benefits, and assistive devices. These data were cross-checked with caregiver reports and verified using institutional documentation. In contrast, the data in Part 2 and Part 3 (care diary) were obtained **directly from caregivers** through face-to-face interviews and represent **primary data**.

Administrative data included information from hospital analytical accounting records (e.g., direct public healthcare costs), disability and educational support services (e.g., special education costs and disability allowances), and clinical documentation such as confirmed diagnosis and GMFCS classification (see Table 1, Part 1). When both caregiver-reported and administrative sources were available for the same variable (e.g., service use, time per visit), administrative data were prioritized due to their standardized and verifiable nature. In cases of discrepancy, the research team resolved inconsistencies through consensus, favoring documented records when reliable, or updated caregiver-reported data when more current or detailed. Data collection spanned two specific time frames: a one-year period for the first and second parts of the questionnaire and a one-month period for the diary corresponding to the third part of the questionnaire (see Table 1).

This study collected data using three main instruments: The Economic Burden Questionnaire of CP (Part 1 and Part 2 in Table 1), a cost diary (mainly to measure productivity loss, Part 3 in Table 1), and the Zarit Burden Interview to capture the subjective burden experienced by primary caregivers (encompasses intangible aspects such as burnout, social constraints, financial strain, emotional distress). This combined approach allowed for a comprehensive evaluation of the direct, and indirect costs associated with the care of children with CP, providing a holistic understanding of the economic and emotional impact on primary caregivers. Table 1 displays the various resource usage categories included in both the questionnaire and the diary. Notably, several modules within Part 1 of the questionnaire (specifically: medical care, conventional treatments, special/adapted education, disability allowance, and mobility aids) were informed by both caregiver report and administrative records. This dual-source structure was designed to reduce recall bias and improve accuracy.

#### 2.2.1 Economic burden questionnaire of CP (EBQ-CP)

The first instrument was a structured questionnaire that included questions on sociodemographic, socioeconomic data, clinical information, and healthcare-related expenses. Costs were categorized into 11 areas: (1) Medical care, (2) Conventional treatments (Therapies and Rehabilitación for CP), (3) Special and adapted education, (4) Disable Allowance, (5) Complementary and Alternative treatments, (6) Special equipment and aids for mobility and communication, (7) Special Clothing, Nutrition and Personal Care, (8) Leisure, Respite Care and Holidays, (9) Transportation, (10) Home modification, (11) Loss of productivity (Assistance and guidance: time spent on care). Primary caregivers reported expenses over the past year for most categories, while for less frequent such as the purchase of adapted vehicles and the purchase or modification of the home. The questionnaire, consisting of 70 questions, was completed through semi-structured interviews of ~30 min, with support from the neuropediatricians and a health economist. The questionnaire also included questions about personal budget resources with a lifespan longer than 1 year. A personal budget is a sum of money, provided by the Spanish government through *disability allowances*, that allows parents to organize and purchase any care, assistance, or support their child needs.

The data collected retrospectively were validated and supplemented by retrieving information from healthcare and government institution records. Healthcare service items were presented as “cost per visit” to facilitate more accurate reporting. Finally, the unit costs were multiplied by the amount of resources used per individual, which provided an estimate of the annual cost per patient by cost type. A note specifying the source (caregiver vs. institutional record) has been added to Table 1 for clarification. For the questionnaire used (see Supplementary material for the complete EQB-CP).

#### 2.2.2 Productivity loss (cost diary)

As part of the productivity loss calculation, the EQB-CP questionnaire included a section on self-reported caregiving time (Table 1, Part 3). This section featured a *care diary* maintained by caregivers for 1 month, providing a detailed record of the daily hours dedicated to the informal care of children with CP.

*Informal care* was defined as any unpaid support provided by the primary caregiver to compensate for the child's disability. Care hours were derived from both questionnaire responses and the monthly caregiving diary, with a maximum limit of 16 daily hours, considering practical caregiving limits and caregiver rest needs. This threshold aligns with prior research on informal caregiving constraints and ethical considerations (29, 30). In our base case, the economic value of informal care was estimated *using the specialized replacement cost method, which is widely accepted as a human capital approac*h, assuming that informal carers provide a quality of care equivalent to that of professional caregivers. Given the high caregiving burden in pediatric CP, this approach better reflects the economic value of unpaid care (16, 27). The hourly cost was estimated using ASPACE's regional reference rates (€17.5/h in 2023) and validated with the Spanish Quarterly Labor Cost Survey 2023, which recorded a similar rate for health and social care services (€17.53/h) (31). This rate reflects the full labor cost from the employer's perspective, including gross wages, social security contributions, and legally mandated benefits.

As part of the sensitivity analysis, we explored an alternative valuation also within the human capital framework. In this scenario, caregiver time was valued as forgone income, using the Spanish minimum wage as a conservative proxy (4, 12).

To obtain an annualized estimate, the *1-month diary data* were extrapolated under the assumption of stable caregiving patterns throughout the year.

#### 2.2.3 Zarit Burden Interview (ZBI)

The third instrument used was the Zarit Burden Interview (ZBI), a globally validated tool for assessing the subjective burden perceived by caregivers. The scale consists of 22 items rated on a 5-point Likert scale (0 = never; 4 = nearly always). The total score ranges from 0 to 88 points, and is usually interpreted as follows: 0–20 points: Not to mild burden; 21–40 points: Mild to moderate burden; 41–60 points: Moderate to severe burden and 61–88 points: Severe burden. The ZBI evaluates multiple dimensions of caregiver burden, including *burnout or physical health, social limitations, financial strain, emotional distress, perception of caregiver demand, and a self-assessment of caregiver performance*. The scale has demonstrated high internal consistency (Cronbach's alpha = 0.93) and test-retest reliability (0.89), with validity confirmed through correlations with other caregiver burden assessment tools (15). Although the ZBI provides valuable insight into the emotional and social burden of caregiving, its results were not used to estimate any cost components in this study.

### 2.3 Ethical approval and consent

The Study of Cerebral Palsy in Pediatric Age in Navarra (EPCINA) received ethical approval from the University Hospital of Navarra's Medical Ethics Committee and was registered with the Clinical Research Secretariat of Navarra (PI_2023/46). Informed consent was obtained in writing from parents, as well as from children aged 12 or older who were capable of understanding the study's purpose. The consent also authorized the collection of personal data.

### 2.4 Cost

A bottom–up approach to cost estimation was adopted, first calculating individual patient costs and then aggregating them to estimate the total population cost across the 11 predefined cost categories. This approach captures real resource utilization at the patient level, providing a more accurate representation of the economic burden. Costs were categorized into three main perspectives: the healthcare system, the government, and families. Costs were estimated in euros for the year 2023.

*Healthcare system costs* encompassed direct medical expenses, including general practitioner, outpatient specialist, diagnostic tests, hospitalization, emergency services, ambulance transfers, prescribed medications, and conventional treatments. Most costs were valued according to Spanish pharmacoeconomic research guidelines, using unit prices (excluding taxes) provided by the University Hospital of Navarra as reported by the analytical accounting department (Table 2) (32). Medication prices were sourced from the public price lists of the Navarra Health Service's Pharmacy Service and Benefits. Outpatient consultation costs were estimated by applying a specialty-specific weighting factor to a baseline price, following university hospital guidelines (32). *Government costs* encompassed expenditures related to disability allowances, special education programs, and financial aid for assistive equipment (e.g., mobility aids and communication devices). These were estimated using official budget allocations and expenditure reports from CREENA and the social services of Navarra for each patient.

**Table 2:** Annual costs per resource unit (euros 2023) for pediatric patients with cerebral palsy (CP).

**Annual cost per resource unit in Euro**							
	**Unit cost**	**Source unit cost**	**Quantity per year child (mean)**	**Source Quantity**	**Quantity per year per child (range)**	**Annual cost (mean)**	**Annual cost (range)**
**CEREBRAL PALSY GENERAL COSTS TYPE**	**Direct health care costs of the public health care system (One-year time period)**						
**General practitioner (per visit)**							
telephone contact	20	1, 2	0.83	2,3	0–1	17	0–20
consultation at the office	35	1, 2	0.85	2,3	0–1	30	0–35
**Outpatient visits, universal rate (per visit)**							
specialist in a university hospital	100	1, 2	2	2	1–2	200	100–200
specialist in a general hospital	85	1, 2	1.18	2	1–3	100	85–255
botulinum toxin treatment	450	2	1	2	0–1	450	0–900
**Outpatient visits at a university (per visit)**							
**Hospital, differentiated rates**							
Neurologist	200	1, 2	1.5	2,3	1–3	300	200–600
Pediatrician	150	1, 2	1.5	2,3	0–3	225	0–450
Rehabilitation specialist	85	1, 2	1.63	2,3	0–4	138	0–340
Ophthalmologist	50	1, 2	1	2,3	0–2	50	0–100
Orthopaedist	70	1, 2	1.5	2,3	0–4	105	0–280
Ear, nose and throat specialist	45	1, 2	1	2,3	0–3	45	0–135
**Diagnostic tests (per test)**							
X-ray hip joints	65	1, 2	0.46	2	0–1	30	0–65
X-ray spinal column	300	1, 2	0.08	2	0–1	24	0–300
MRI-spinal column	250	1, 2	0.16	2	0–1	40	0–250
MRI-knee	75	1, 2	0.33	2	0–1	25	0–75
Electroencephalogram	150	1, 2	0.23	2	0–1	35	0–150
Barium swallowing test	100	1, 2	0.35	2	0–1	35	0–100
Salivary measurement	200	1, 2	0.15	2	0–1	30	0–200
**Conventional Medicine (per visit/session)**							
**Therapy and rehabilitation**							
Speech therapist	25	1, 2,4	32	3,5	0–98	800	0–2450
Physiotherapist	30	1, 2,4	64	3,5	0–144	1,920	0–4320
Occupational therapist	25.58	1, 2,4	22	3,5	0–96	562.8	0–2456
**Treatments directly related to CP comorbidities (per visit)**							
Epilepsy	150	1,4	2.33	2,3	1–4	350	150–600
Intellectual Impairment	80	1,4	1.88	2,3	1–3	150	80–240
Visual Impairment	50	1,4	1	2,3	0–1	50	0–50
Hearing Impairment	45	1,4	1.67	2,3	1–3	75	45–135
Speech Impairment	70	1,4	4.83	2,3	1–12	339	70–840
**Hospitalization**							
University hospital per day	500	1,2,4	1	2	0–3	500	0–1500
Ambulance travel	150	2,4	1.33	2	1–2	199.5	150–300
	**Unit cost**	**Source unit cost**	**Quantity per year child (mean)**	**Source Quantity**	**Quantity per year per child (range)**	**Annual cost (mean)**	**Annual cost (range)**
**Prescribed medication (By prescription)**							
CP and Comorbidities	250	4,7	4.04	2,3	1–12	1010.6	250–3000
**Special equipment and aids for mobility (per item)**							
Wheelchair, splints, standing frame, etc)	3000	1,4	1.6	2,3	1–3	4800	3000–9000
Other equipment's and aids	850	1,4	1.265	2,3	1–3	1075.2	850–2550
**Special or adapted education**+							
Teacher specialized in therapeutic pedagogy (1 for every 8 children)	30,000	4,5,6	0.125	3,5	0.125–0.2	3750	3750–6000
Caregiver specialized in educational support (1 for every 5 children)	15,876	4,5,6	0.2	3,5	0.2–0.25	3175	3175–3969
Special adapted transportation	850	4,5,6	0.85	3,5	0–1.5	722	0–1275
Costs of specialized learning equipment	1,256	4,5,6	0.8	3,5	0–1	1004.8	0–1256
Costs of specialized learning material and excursions fee per year	281	4,5,6	1	3,5	1	281	281
**Out-of-pocket costs**							
**Complementary Medicine (per visit/session)**							
Speech therapy	50	6	30	3	0–60	1500	0–3000
Motor physiotherapy	50	6	50	3	0–100	2500	0–5000
Occupational therapy	45	6	10.4	3	0–20	468	0–900
**Alternative medicine (per visit/session)**							
Optometrist	70	3	2.19	3	0–2	153	0–140
Therasuit	80	3	11.25	3	1–37	900	80–2960
Peto Method	60	3, 6	22.5	3	10–40	1350	600–2400
Osteopathy	85	3	1	3	0–1	85	0–85
Homeopathy	80	3	1.06	3	0–2	85	0–160
**Special equipment and aids that are not covered or not frequently provided. (per Item)**							
Communication equipment	200	1,4	0.2	3	0–1	40	0–200
Mobility equipment	500	1,4	0.32	3	0–2	160	0–1000
**Clothing, Nutrition and Personal Care (per purchase)**							
Specialized clothing	200	3	1.5	3	1–4	300	200–800
Specialized nutrition	100	3	1.51	3	1–3	151	100–300
Specialized personal care incontinence products	150	3	1.33	3	1–2	200	150–300
**Subscriptions to patients' associations** ^*^	200	3, 6	1	3	0–1	200	0–200
**Leisure, Respite Care and Holidays (per activity, per times)**							
Leisure or sport	150	3	1.13	3	1–2	170	150–300
Respite care	180	3,6	0.92	3	1–2	166	180–360
	**Unit cost**	**Source unit cost**	**Quantity per year child (mean)**	**Source Quantity**	**Quantity per year per child (range)**	**Annual cost (mean)**	**Annual cost (range)**
Holidays	200	3	1.25	3	1–2	250	200–400
**Transportation and fuel**							
Price per kilometer (by car)	0.35	1, 3	400	3	80–800	140	28–280
Public transport (Pass per month)	14.55	1, 3	6	3	0–12	87.3	0–174.6
Tolls/parking (per use/time)	2.10	1, 3	57.14	3	1–124	120	2,10–260
**Wheelchair van/vehicles and adaptations**							
Car purchase (5 years)^*^	35,000	1, 3	0.42	3	0–1	14700	0–14,700
Car modifications (5 years)	12,000	1, 3	0.20	3	0–1	2400	0–12,000
Other car-related costs (5 years)	3000	1, 3	0.20	3	0–1	604.7	0–3000
**Housing and modifications (5 years)**							
Costs of moving location^*^	80,000	1,3	0.2	3	0–1	16000	0–80,000
Home access modifications	5500	3	0.145	3	0–1	800	0–5500
Internal home modifications	1500	3	1.33	3	1–3	2000	1–4500
Home equipment	450	3	0.89	3	0–1	404.5	0–450

**Sources:** (1) Spanish guideline for PharmacoEconomics research, (2) Data University Hospital Navarra, (3) Patient questionnaire, (4) Public rates BON, (5) CREENA, (6) ASPACE, (7) Pharmacy and Benefits Service of the Navarra Health Service.

^*^Estimation of infrequent out-of-pocket costs. + Extra cost in education compared to the pediatric age of the general population. Note: Some values have been rounded.

*Family-borne costs* included mainly out-of-pocket expenses for complementary and alternative treatments, specialized equipment for mobility and communication that is not often covered, transportation, home modifications, and productivity losses due to caregiver responsibilities. Productivity losses were calculated using the replacement cost method, due to the high demand for care in the pediatric age group. Transport costs were limited to travel to and from the hospital, the center for therapy sessions, and the educational center. The total cost of the wheelchair accessible vans was calculated individually for each patient, depending on whether the van was used exclusively for the patient or also for general family purposes, with specific calculations for wheelchair-accessible vans, insurance, maintenance, and road tax (Table 2). Where families used the wheelchair vans for general purposes, the acquisition costs were adjusted for the cost of a standard mid-range car according to the Spanish Consumers” Organization Guide, August 2023. In these cases, insurance, maintenance, and road tax costs were not included.

Although healthcare cost data are typically right-skewed, the arithmetic mean was used as the primary measure of central tendency for individual and aggregated costs (24, 25). This choice is consistent with the methodological standards in cost-of-illness studies, as the mean allows for the estimation of total population-level costs and facilitates national extrapolation and economic modeling (26, 33). While median values are more robust to outliers, they are not additive and therefore unsuitable for calculating the total economic burden (34). To address the inherent skewness and variability in the data, additional analyses based on empirical percentiles and log-transformed confidence intervals were included, as described in subsequent sections.

#### 2.4.1 Statistical approach to uncertainty

To estimate the variability and robustness of cost results, we applied two complementary approaches consistent with best practices in COI studies involving skewed and heterogeneous cost data (34, 35).

First, we performed *a scenario-based sensitivity analysis* using empirical 5th and 95th percentiles of observed annual societal costs by resource category to define plausible best-case and worst-case scenarios (25). This non-parametric approach avoids relying on extreme minimum and maximum values that could bias the results (36). It is particularly appropriate in conditions like pediatric CP, which exhibit high variability and asymmetric cost distributions. These scenarios provide decision-makers with a realistic range of possible economic outcomes (10).

Second, we estimated 95% *confidence intervals (CI)* for the ratio of mean total societal costs *between severity groups (GMFCS I–II vs. III–V)* using a log-transformation of the ratio of means. Group-specific means and standard deviations were calculated, and the standard error of the log-ratio was derived using the delta method under the assumption of independence between groups. The resulting CI were then back-transformed to the original scale. This method is widely used in economic evaluations of skewed cost data because it reduces the impact of outliers and produces robust, interpretable estimates for subgroup comparisons (37).

Data management and all analyses were performed with Microsoft Excel. Statistical analyses were performed with R software version 4.3.2 (38).

## 3 Results

A total of 148 children aged between 3 and 18 years (mean age: 9.72 ± 4.22 years) were included in the study. The distribution by Gross Motor Function Classification System (GMFCS) levels was as follows: 37 children at level I, 33 at level II, 16 at level III, 21 at level IV, and 41 at level V. The majority (79%) of participants had spastic cerebral palsy. Notably, the response rate for the questionnaires and diaries was 100%, ensuring complete data availability. Participant characteristics for the entire population are presented in Table 3.

**Table 3:** Sociodemographic, clinical, and socioeconomic characteristics of Cerebral Palsy patients.

**Type of variable**	**Mean (SD) or *N* (%)**
***Sociodemographic***	
***Age***	***Mean (SD)***
*Age of caregiver*	42.97 (6.25)
*Age of child with CP*	9.72 (4.22)
***Age range of children***	***N (%)***
Age 3–6	46 (31%)
Age 7–12	60 (41%)
Age 13–18	42 (28%)
***Gender of child with CP***	***N (%)***
Female	70 (47%)
Male	78 (53%)
***Gender of Caregiver***	***N (%)***
Female	98 (66%)
Male	50 (34%)
***Caregiver's marital status***	***N (%)***
Single	24 (16%)
Married	106 (72%)
Divorced	18 (12%)
**CLINICAL**	
**Cerebral Palsy Motor Type(s)** ^*^	***N (%)***
Spastic	117 (79%)
Ataxic	8 (5.40%)
Dyskinetic	8 (5.40%)
Mixed	15 (10.81%)
**GMFCS Level** ^*^	***N (%)***
Level I	37 (25%)
Level II	33 (22.29%)
Level III	16 (10.81%)
Level IV	21 (14.18%)
Level V	41 (27.70%)
Missing	0 (0%)
Level I-II (Independent walker)	70 (47.29%)
Level III-V (Walker with aid or wheelchair)	78 (52.70%)
**Comorbidities associated with CP** ^*^	***N (%)***
No other comorbidities	26 (17.56%)
Epilepsy	61 (41.21%)
Intellectual impairment	65 (43.91%)
Visual impairment	62 (14.86%)
Hearing impairment	22 (32.42%)
Speech impairment	75 (50.67%)
ADHD	35 (23.64%)
ASD	17 (11.48%)
Mental health condition	60 (40.54%)
Other comorbidities	37 (25%)
**SOCIOECONOMIC**	
***Highest Level of Education of Carer***	***N (%)***
Elementary education	15 (10.13%)
Secondary education	25 (16.89%)
Tertiary education	108 (72.97%)
Other/none	0 (0%)
Missing/unknown	0 (%)
***Household Income Before Tax***	***N (%)***
Less or up €30,000 per year	28 (18.91%)
€31,000–€52,000 per year	61 (41.21%)
€53,000–€72,000 per year	49 (33.13%)
More than €73,000 per year	10 (6.75%)
Did not wish to disclose	0 (0%)
***Leaves work for son/daughter's CP***	**N (%)**
Yes	73 (49.33%)
No	75 (50.67%)
**Place of residence (Access to health services)**	***N (%)***
Easy access (hospital and rehabilitation center <30 min away)	80 (54%)
Limited access (specialized services between 30–90 min away)	40 (27%)
No local access (more than 90)	28 (19%)

^*^Types of CP correspond to the expected prevalence by type, according to the SCPE. ^*^Some children may present more than one comorbidity associated with CP, for this reason the N and (%) in the category (comorbidities associated with CP) exceed the total N of the study population. ^*^GMFCS level: The Gross Motor Function Classification System for CP is based on patient self-initiated movement with emphasis on sitting (trunk control), transfers and mobility.

### 3.1 Caregiver profile and perceived burden (Zarit Burden Interview)

**Figure 1:**
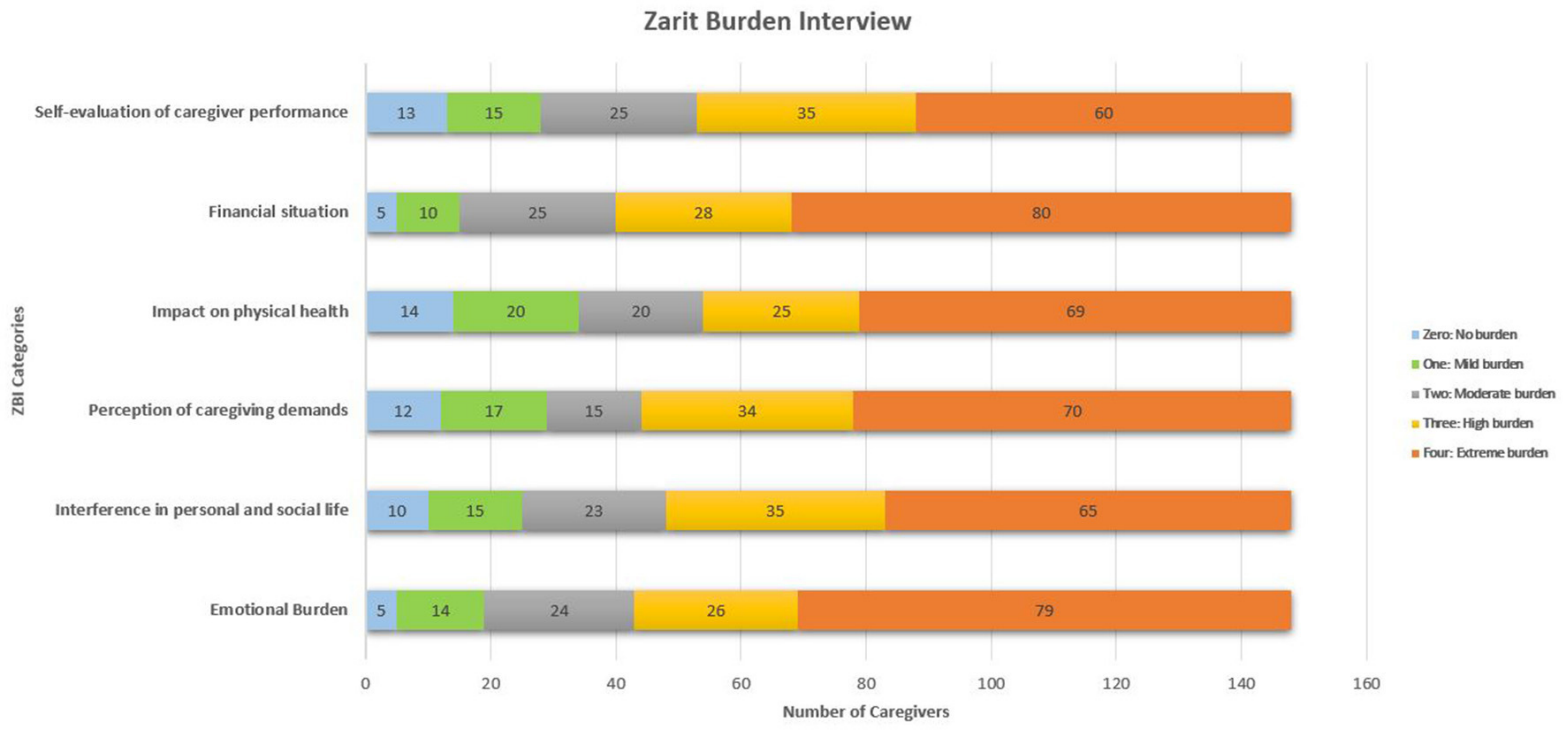
Distribution of caregiver responses across six burden-related categories of the Zarit Burden Interview (ZBI). Each **horizontal bar** represents the number of caregivers **(*N*)** who selected each response category on a 5-point Likert scale, where: 0 = No burden, 1 = Mild burden, 2 = Moderate burden, 3 = High burden, and 4 = Extreme burden. The bars are **color-coded** for each Likert level and grouped by burden domains (e.g., emotional stress, social life, physical strain). This visualization highlights the distribution and intensity of perceived burden across relevant caregiving dimensions.

A total of 148 caregivers were included in the study, of which 98 were women (66%) and 50 were men (34%) with a female preponderance in caregiving. The characteristics of the parents were analyzed in terms of age, sex, and educational level. University education was more prevalent among mothers (45.27%, *n* = 67) than fathers (27.70%, *n* = 41). Additionally, a significant proportion of mothers (37.16%, *n* = 55) left their jobs to care for their children, while this was the case for only 12.16% (*n* = 18) of fathers. We found that 48% (*n* = 71) of caregivers experienced severe burden 45% (*n* = 66) of caregivers had moderate to severe burden and 7% (*n* = 11) had mild to moderate burden. Besides, Figure 1 shows us that 44% of caregivers indicated that their social life had -almost always- been affected. 53.37 % of the caregivers most frequently had a feeling of emotional burden due to stress and uncertainty about the future. 46.62 % of the caregivers were worried about their physical health most of the time. Many of the health issues mentioned included back pain, arm pain, and hypertension. Around 35% of the caregivers had chronic back pain from carrying their children around. Finally, 54% of caregivers were concerned about not having enough money to have a decent life and to be able to provide more treatment opportunities for their children.

### 3.2 Costs from the perspectives of the health system, government, and families

Table 2 shows the different resources, including their unit costs, usage quantities, and total annual costs per resource. The societal costs can be subdivided into costs of the health system, the government, and the families (Table 4). From the *perspective of the health system*, mean annual costs per child were estimated at €8,806. The highest costs fell into the categories of medical care (€3,702; 42.03%). From the *perspective of the government*, mean annual costs were estimated at €18,104 of which €8,082.9 (44.64%) for special and adapted education. From the *family perspective*, mean annual costs were estimated at €75,225 of which €60,638 (80.63%) for Assistance and guidance (time spent on care), and complementary and alternative treatments represent €7,041 (9.36%).

**Table 4:** Mean annual costs by resource category from the perspectives of the healthcare system, government, and the families (euros 2023).

**Mean annual costs per resource category from the perspectives of the health system, the government and families (in Euro)**						
**Resource category**	**Healthcare perspective**		**Governmental perspective**		**Family perspective**	
	**Mean costs**	**% of total**	**Mean costs**	**% of total**	**Mean costs**	**% of total**
**Medical care**	**3,702**	**42.02**	**0**	**0.00**	**101.6**	**0,13**
General practitioner care	47	0.53	0	0.00	0	0.00
Specialist medical care	863	9.79	0	0.00	0	0.00
Tests	219	2.48	0	0.00	0	0.00
Hospitalization	700	7.94	0	0.00	0	0.00
Treatments related to comorbidities associated with pc	964	10.94	0	0.00	0	0.00
Medication	909	10.32	0	0.00	101.6	0.13
**Conventional treatments**	**3,282.8**	**37.27**	**0**	**0.00**	**0**	**0.00**
**Special and adapted education**	**0**	**0.00**	**8,082.9**	**44.64**	**850**	**1.12**
Cost of formal care, learning and adapted spaces	0	0.00	8,082.9	44.64	850	1.12
**Disability allowance**	**0**	**0.00**	**4,569.6**	**25.24**	**0**	**0.00**
Personal budget	0	0.00	4,569.6	25.24	0	0.00
**Complementary and Alternative treatments**	**0**	**0.00**	**0**	**0.00**	**7,041**	**9.35**
Rehabilitación	0	0.00	0	0.00	4,468	5.93
Alternative medicine	0	0.00	0	0.00	2,573	3.42
**Special equipment and aids for mobility and communication**	**1,822**	**20.69**	**4,052.6**	**22.38**	**200**	**0.26**
Wheelchairs	1,440	16.34	3,287.52	18.15	160	0.21
Other equipment and aids	382	4.33	765.12	4.22	40	0.05
**Special clothing, nutrition and personal care**	**0**	**0.00**	**200**	**1.10**	**451.4**	**0.60**
General Personal Care (N+C)	0	0.00	0	0.00	451.4	0.60
Incontinence products	0	0.00	200	1.10	0	0.00
**Leisure, respite care and holidays**	**0**	**0.00**	**0**	**0.00**	**586.3**	**0.77**
**Transportation**	**0**	0.00	**1,200**	**6.62**	**2,152.7**	**2.86**
Van/Purchase or modification of vehicles	**0**	0.00	1,200	6.62	1,352	1.79
Traveling expenses	0	0.00	0	0.00	800.7	1.06
**Home modification**	**0**	**0.00**	**0**	**0.00**	**3,204.5**	**4.25**
**Loss of caregiver productivity**	**0**	**0.00**	**0**	**0.00**	**60,638**	**80.60**
Assistance and guidance: time spent on care	0	0.00	0	0.00	60,638	80.60
**Total of mean annual costs**	**8,806**	**100.00**	**18,104**	**100.00**	**75,225**	**100.00**

Bold text indicates the higher-level groupings of resources.

### 3.3 Costs from a societal perspective

Table 5 shows the annual costs to society distributed across the 11 main resource categories. From a societal perspective, the mean annual costs were €102,135. The highest expenditures were for loss of caregiver productivity which include assistance and guidance: time spent on care (€60,638; 59.37%), followed by special and adapted education (€8,932; 8.75%), out-of-pocket expenses including alternative and complementary therapies not covered by the health care system (€7,041; 6.89%), special equipment and aids for mobility and communication (€6,075; 5.95%), and transportation costs (€3,352; 3.28%). The costs within the category of medical care were relatively low accounting for only (3,803; 3.72%) of total costs.

**Table 5:** Mean Annual Costs by resource category from the societal perspective (euros 2023), including results from sensitivity analyses.

**Mean annual costs per resource category from the societal perspective (in Euro), including the results of the sensitivity analyses**						
**Resource category**	**Base case scenario**		**Sensitivity analysis in possible scenarios**			
	**Mean costs**	**% of total**	**Mean costs**	**% of total**	**5th percentile cost (best case)**	**95th percentile cost (worst case)**
**Medical care**	**3,803.6**	**3.72**			**2,759**	**7,039**
General practitioner care	47	0.04				
Tests	219	0.21				
Hospitalization	700	0.68				
Treatments related to comorbidities associated with CP	964	0.94				
**Specialist medical care**						
Differentiated rates (base)^*^	863	0.84				
Universal rates^*^			750	0.73		
Medication	1,010.6	0.99				
**Conventional treatments**	**3,282.8**	**3.21**			**1,200**	**5,000**
Therapies and Rehabilitation for CP	3,282.8	3.21				
**Special and adapted education**	**8,932.9**	**8.74**			**4,500**	**15,000**
Formal care, learning, and adapted spaces	8,932.9	8.74				
**Disability Allowance**	**4,569.6**	**4.47**			**0**	**8,005**
- Personal budget year 1 (base)	4,569.6	4.47				
- Personal budget year 2			4,750	4.65		
**Complementary and Alternative treatments**	**7,041**	**6.89**			**0**	**16,825**
Rehabilitation	4,468	4.37				
Alternative medicine	2,573	2.51				
**Special equipment and aids**	**6,075.2**	**5.94**			**1,250**	**12,000**
Wheelchairs	4,800	4.69				
Other equipment and aids	1,275.2	1.24				
- Depreciation 5 years (base)	6,075.2	5.95				
- Depreciation 3 years			4,252.6	4.16		
- Depreciation 7 years			1,822.5	1.78		
**Special clothing, nutrition and care**	**651.4**	**0.63**			**209**	**1,250**
General personal care (nutrition and clothing)	451.4	0.44				
**Resource category**	**Base case scenario**		**Sensitivity analysis in possible scenarios**			
	**Mean costs**	**% of total**	**Mean costs**	**% of total**	**5th percentile cost (best case)**	**95th percentile cost (worst case)**
Incontinence products	200	0.19				
**Leisure, respite care and holidays**	**586.3**	**0.57**			**200**	**977.5**
**Transportation and vehicles modifications**	**3,352.7**	**3.28**			**650**	**5,000**
- Van depreciation 7 years (base)	2,400.7	2.35				
- Van depreciation 5 years			1,822.6	1.78		
- Van depreciation 9 years			3,281.7	3.21		
Traveling expenses	952	0.93				
**Home modification**	**3,204.5**	**3.18**			**0**	**12,000**
- Depreciation 10 years (base)	3,204.5	3.18				
- Depreciation 15 years			4,806.7	4.70		
**Loss of caregiver productivity** ^*^	**60,638**	**59.37**			**25,550**	**102,200**
Caregiver assistance and guidance	60,638	59.37				
**Total of mean annual costs**	€**102,135**	**100**			€**36,318**	€**185,296**

“Mean Costs” refer to the base case scenario and represent the arithmetic average of annual costs across all individuals in the sample “Percentile” values were calculated using the empirical distribution of individual annual costs per category (including zero values). The total cost values shown in the last row of the table (€36,318 and €185,296.5) were obtained by summing the 5th and 95th percentile values across all cost categories.

^*^Based on the replacement cost method.

Within the category of equipment and special aids, the purchase, adaptations, and maintenance of wheelchairs accounted for 74% of the costs. All of the children in motor level GMFCS III-V had a non-powered wheelchair. In addition, 19 of them also had a power wheelchair. Within the category of therapies and rehabilitation*, n* = *94 children received alternative therapies* (Peto Method, Therasuit, and Equine Therapy) *and complementary therapies* to those they already received in the public health system (Rehabilitation and Speech Therapy). On the other hand, the rest of the children *n* = *54 only received the conventional therapies offered by the public health* system, since the family did not have the resources for extra therapies. Of these 54 families, only *7 thought that their child had enough with what the public health system* provided in this type of treatment. In the category of transportation expenses, wheelchair vans accounted for 97% of the expenses.

### 3.4 Productivity loss

The mean cost per patient of informal care for pediatric CP was estimated to be €60,630 in 2023 (see Table 5), with patients in GMFCS levels IV and V accounting for 53.09% of the total expenditure. Within the category of lost caregiver productivity, GMFCS disability level I had a mean cost of €45,920, while GMFCS II was €48,196, compared to €57,487 for GMFCS disability level III, €73,304 for GMFCS IV, and €78,675 for GMFCS V (data not shown).

The intensity of informal care varied substantially by disability level. While the mean number of hours of care per child was 9.5 h/day (3,465 h/year), this ranged from 7.2 h/day in GMFCS I to 12.3 h/day in GMFCS V, indicating a sharp increase in caregiver burden at higher disability levels. The variability was particularly high in GMFCS III (SD = 4.08 h), suggesting substantial differences in care needs within this group (data not shown). These findings highlight the progressive nature of care dependency in pediatric CP and its significant impact on caregivers” productivity losses.

### 3.5 Sensitivity analyses

To assess uncertainty in annual costs from a societal perspective, we conducted sensitivity analyses using two complementary approaches. First, we estimated optimistic (Best Case) and pessimistic (Worst Case) scenarios based on the 5th and 95th percentiles of the individual distribution of annual costs per resource category (Table 5). These percentiles represent the values below which 5% and 95% of the sample observations fall, respectively. This approach captures the variability in costs across individuals while limiting the influence of extreme outliers and measurement errors, an established method in economic evaluation (25, 26). Table 5 presents these results in the last two columns, labeled “5th Percentile Cost (Best Case) and 95th Percentile Cost (Worst Case). In contrast, the “Mean Costs” column refers exclusively to the base case scenario and was calculated as the arithmetic average of annual costs across all individuals in the sample (*n* = 148).

The values for the 5th and 95th percentiles were calculated directly from the empirical distribution of individual costs by category, including observations with zero cost. No averages were calculated for subgroups. To estimate the total annual costs in the best- and worst-case scenarios, these percentiles were added together across all categories (see the last row of the table). This procedure provides a plausible range of total costs per individual, ranging from €36,318 to €185,296.5, reflecting patterns of systematically low or high resource use. This variation is mainly driven by the degree of gross motor functional dependence (GMFCS), where children with higher impairment levels incur increased costs due to greater caregiver burden, more intensive rehabilitation needs, and additional special education support. These findings highlight the importance of considering a range of cost scenarios when planning resource allocation and designing support policies.

In addition, based on the statistical approach outlined in Section 2.4.1, we performed a stratified cost analysis to examine differences in societal burden according to motor severity, using the GMFCS level as the primary stratification variable. Children classified as having severe functional limitations (GMFCS levels III–V) incurred nearly *twice the mean annual societal costs* compared to those with milder impairments (GMFCS I–II), with a cost ratio of *1.96 (95% CI: 1.92–2.01)*. This substantial gap underscores the significantly higher care demands, intensity of rehabilitation, and need for special education resources in children with greater motor disability (3). These findings highlight *GMFCS as a key cost driver* and complement the percentile-based uncertainty analysis presented in Table 5.

**Figure 2:**
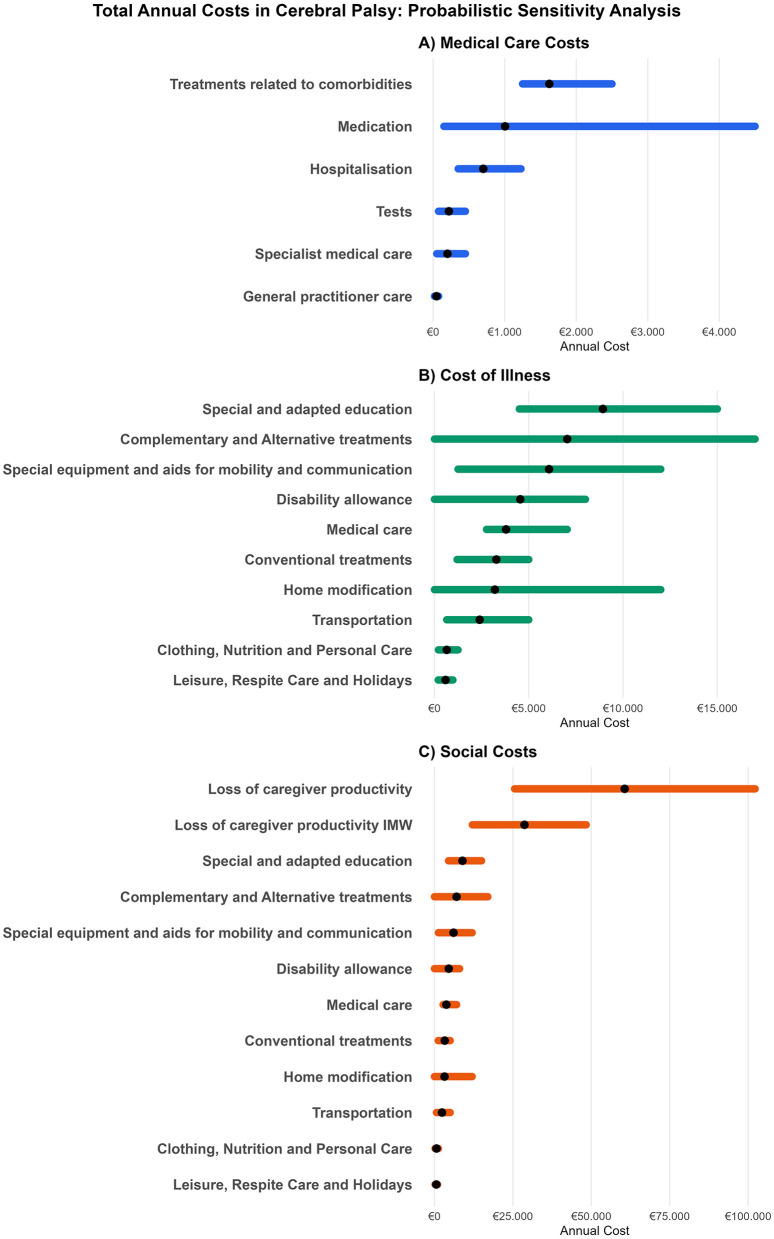
Sensitivity analysis from different perspectives: **(A)** Medical Care; **(B)** Cost of Illness (COI); **(C)** Social Cost (euros 2023). Tornado plots showing the uncertainty range (5th−95th percentiles) and mean values of annual costs across three domains: **(A–C)** represent progressively broader cost categories: **(A)** Medical Care Costs include direct health expenditures only; **(B)** Cost of Illness includes medical costs plus education, alternative therapies, assistive devices, allowances, transport, and other non-medical costs; **(C)** Social Cost includes all COI components plus productivity losses due to informal caregiving, reflecting the full societal burden. **Horizontal bars** represent the 90% uncertainty intervals derived from probabilistic sensitivity analysis, and the **black dots** indicate the mean cost per category. All costs are presented in 2023 euros (€); **(C)** Displays the **alternative scenario** using the **Interprofessional Minimum Wage (IMW)** to value informal caregiver time, illustrating the sensitivity of societal cost estimates to the valuation approach.

Furthermore, we analyzed variations in caregiving intensity over time and compared caregiver productivity loss estimates using two valuation methods: replacement cost (€17.5/h) and foregone earnings based on Spain's minimum wage (IMW) in 2023 (€8.28/h). Two scenarios were defined: (1) a base case using the replacement cost method and (2) an alternative more conservative scenario, assuming that the caregiver would earn at least the IMW. In the base case, productivity loss ranged from €25,550 (5th percentile) to €102,200 (95th percentile) when using the replacement cost of a specialized professional. In the alternative scenario, where the IMW-based valuation was applied, productivity loss varied between €12,088.8 (5th percentile) and €48,355.2 (95th percentile) (Figure 2C). These results highlight the significant impact of valuation methods on cost estimates. When recalculating total societal costs using the IMW instead of the skilled replacement cost, the mean annual cost per child decreased from €102,135 to €70,190. This underscores the high sensitivity of societal cost estimates to the method chosen for valuing caregiver time.

As shown in Figure 2A, the highest medical care costs are associated with treatments for comorbidities, while medication expenses exhibit the greatest variability, disproportionately affecting children requiring multiple medications, particularly when copayments are low. Regarding COI, Figure 2B the highest expenditures correspond to special and adapted education, as well as complementary and alternative treatments, primarily funded by the government and families. Both categories display substantial variability among patients, highlighting disparities in access in these categories likely caused by high financial burdens.

### 3.6 National-level cost extrapolation

Although this study is regionally based, we conducted a national-level extrapolation to estimate the broader economic implications of pediatric CP in Spain. Based on official population data from the Spanish Statistical Office (INE), an estimated *7.43 million children aged 3–18* resided in Spain in 2023 (39). Applying a conservative CP prevalence of *2 per 1,000 children* (19), we estimated a national pediatric CP population of *14,862 children*. Using the GMFCS-level distribution observed in our sample and the corresponding stratified mean annual costs, we estimated a *total annual societal cost* of ~€*1.52 billion*, and a *Cost of Illness* of €*616.4 million*. Additionally, *direct medical care costs* alone were estimated at €*56.4 million* annually. These data underscore the disproportionate economic burden placed on families and social systems (14). Children classified at *GMFCS levels IV and V*, who represent around *42% of the CP population*, account for *more than half of the total societal costs*, reflecting the escalating needs associated with higher levels of functional impairment (11). A detailed breakdown of these national-level estimates is provided in Table 6.

**Table 6:** Estimated national annual societal costs, costs of illness, and medical care costs (euros, 2023) of pediatric cerebral palsy in Spain, stratified by GMFCS level.

**GMFCS level**	**% of CP population**	**Estimated *N* of children**	**Mean social cost (€)**	**Mean cost of illness (€)**	**Mean cost of medical care (€)**	**Total, social cost (€M)**	**Total cost of illness (€M)**	**Total cost of medical care (€M)**
I	25.00%	3,715	77,341.00	31,420.00	3,507.00	287.30	116.80	13.04
II	22.29%	3,311	80,533.00	32,336.00	3,685.00	266.70	107.10	12.20
III	10.81%	1,607	101,758.00	44,270.00	3,684.00	163.60	71.10	5.92
IV	14.18%	2,105	122,470.00	49,166.00	3,889.00	257.90	103.50	8.19
V	27.70%	4,124	131,537.00	52,862.00	4,141.00	542.50	217.90	17.09
**Total**	**100.00%**	**14,862**	–	–	–	**1,517.90**	**616.40**	**56.44**

**Sources:** Population aged 3–18: Instituto Nacional de Estadística (INE), 2023; CP prevalence: 2 per 1,000 children (SCPE, 2019); Cost inputs per GMFCS level: EPCINA Study, Navarra (2023–2024). Total annual costs associated with cerebral palsy are categorized into three components: **(A)** Medical Care Costs: include hospitalisation, outpatient specialist and general practitioner visits, diagnostic tests, medication, and treatments for CP comorbidities. **(B)** Cost of Illness (COI): includes all items under Medical Care plus special/adapted education, complementary and alternative treatments, special equipment and mobility aids, disability allowance, home modifications, transportation, special clothing, nutrition and personal care, and leisure or respite care. **(C)** Total Social Cost: adds the estimated productivity loss of informal caregivers to the COI, representing the full economic impact from a societal perspective. All cost categories are mutually exclusive and additive. Values are presented in 2023 euros (€), using English numeric notation (comma as thousand separators, period as decimal point).

## 4 Discussion

In this economic burden study based on COI methodology, we calculated and classified costs. We estimate that the mean annual cost per child with CP from a societal perspective amounts to €102,135, exceeding Spain's per capita healthcare expenditure in 2023 (€2,805) by more than thirty times (40). Although it would be methodologically preferable to compare healthcare costs with those of children without CP in the same age group, there are no publicly available data in Spain disaggregated by age group that would allow for an estimate of average annual healthcare expenditure for the general pediatric population (39, 40). Consequently, the per capita healthcare expenditure was used as a proxy comparator, in line with the methodology adopted by previous international COI studies (4, 9, 14). While this approach may underestimate the differential with respect to the pediatric population, it nonetheless provides a useful benchmark to contextualize the considerable magnitude of the economic burden.

This economic burden falls mainly on families (73.66%), followed by the government (17.72%) and the healthcare system (8.62%). These findings highlight the magnitude of CP's economic impact (4, 11, 14), underlining the need for public policies that comprehensively address the needs of affected families, particularly regarding the caregiving burden. When the valuation of caregiver time was based on Spain's interprofessional minimum wage (IMW) instead of a specialized replacement cost, the average societal cost per child decreased from €102,135 to €70,190. This substantial difference illustrates how strongly cost estimates depend on the chosen method for valuing informal care, and underscores the need for transparent reporting of both conservative and comprehensive scenarios in future analyses. Because specialized care is required on an ongoing and permanent basis due to the nature of the disease, we consider the base-case estimate (€102,135, using specialized replacement cost) to be the most realistic scenario (27, 41, 42). However, in a scenario excluding caregiver productivity loss, the COI would be €41,497 with the burden distributed more equally between the government (43.62%), families (35.15%), and the public healthcare system (21,23%), although families would still bear a substantial economic strain.

When considering the social cost scenario of CP, our results are comparable to those reported in Australian studies, where estimated social costs reached €90,597 in 2018 (14). Similar findings have been documented in Canada (€94,000) (18); however, in these countries, the mean cost per household was significantly lower due to greater state support (11, 14). This difference may be attributed to variations in healthcare financing models and social support systems. In this respect, our comparison suggests that Spain has a high reliance on informal care, which increases the financial burden on families (13, 16, 43). On the other hand, when considering the scenario that excludes productivity loss, our results also align with the European context. For example, a Dutch study conducted in 2010 estimated annual CP-related costs of up to €40,265 per patient (9). Unlike other countries, where public interventions cover a significant portion of therapeutic needs (4, 9, 14), in Spain, private spending on therapies and specialized equipment represents a major barrier for families (11, 29, 30).

Based on interviews with 148 caregivers of patients with CP, the economic burden from a social perspective is substantial for both families and society. The main component of social costs was the loss of productivity among primary caregivers, accounting for 59.37% of the total. The high level of caregiver commitment, with a mean of 9.5 h per day (data not shown), highlights the inadequacy of formal support services (44). Additionally, out-of-pocket expenses for complementary and alternative treatments were significant, representing 6.89% of the total cost (11, 45). Several factors contribute to this economic burden, including prolonged dependence on caregiver support, the progressive deterioration of motor function, and the recurrent use of rehabilitation services (4). These findings emphasize the need to recognize the unpaid work of informal caregivers, as acknowledging their contribution is essential for designing policies that provide financial compensation or support programs to alleviate their burden. In this study, caregiver time was valued using full labor cost estimates, which include gross wages, social security contributions, and legally required benefits. This provides a realistic basis for policy design and caregiver compensation.

Beyond financial costs, the impact of CP extends to the psychological and social burden on caregivers. The Zarit Burden Interview revealed that nearly half of the caregivers experienced severe burdens, highlighting the considerable emotional and physical strain associated with long-term caregiving. These findings align with previous research emphasizing the high prevalence of stress, anxiety, and social isolation among caregivers of children with chronic conditions (27, 42, 43). Recognizing this burden is essential for developing policies that not only provide financial compensation but also ensure access to psychological and social support programs tailored to caregivers” needs.

Several countries have implemented effective policies to alleviate the burden on caregivers of children with disabilities, offering models that Spain could adopt. For example, Australia funds early intervention programs to improve access to therapies and assistive technologies, enhancing long-term outcomes and reducing healthcare costs (10, 11, 14). Similarly, Canada subsidizes respite care programs, easing caregiver strain and improving wellbeing (18). To reduce the financial burden on families and ensure more equitable access to care in Spain, the following actions are essential: First, expanding publicly funded respite care programs to prevent caregiver burnout and reduce reliance on emergency services. Second, it is essential to increase public coverage of essential therapies and assistive devices, such as physical therapy, occupational therapy, and speech therapy, which have demonstrated clinical efficacy across all levels of severity (46, 47). However, their availability and intensity are often limited, especially in milder cases. This approach must ensure not only the availability of and access to these therapies, but also the personalization of treatment, adapting it to the specific functional needs of each child to maximize its effectiveness.

Third, reorganizing public healthcare funding involves improving the planning, allocation, and coordination of services for cerebral palsy (CP). Although Spain's healthcare system is universal, challenges remain in providing adequate and equitable care for children with complex chronic conditions. Public coverage guarantees only a minimum level of services, constrained by budget limits and competing health priorities. There is no national CP registry or standardized treatment protocols based on severity (e.g., GMFCS), leading to regional and clinical disparities that affect both severe and mild cases, with the latter often overlooked. After childhood, many patients lose access to structured support and rely on private long-term or residential care, especially in the absence of family caregivers. This reflects the high burden of informal care, which accounts for a substantial share of the total cost. Thus, reorganizing funding requires prioritizing needs-based care planning and ensuring continuity across the life course, supported by evidence on costs, effectiveness, and cost-effectiveness of interventions. Fourth, it is essential that the State guarantees and finances comprehensive prevention and rehabilitation services within a structural policy aimed at reducing the long-term burden faced by patients and their families (10, 23). This broad and systemic approach seeks to reduce inequalities and facilitate equitable and sustained access to preventive and rehabilitative interventions, overcoming *ad hoc* and fragmented provision.

Several methodological strengths underpin the robustness of our results and reinforce the study's contribution to understanding the economic impact of pediatric CP. First, it adopts a comprehensive social perspective by capturing both direct and indirect costs, including caregiver burden, providing a complete view of how the economic impact is distributed across the healthcare system, government, and families (24). Second, the estimation of the regional prevalence of pediatric CP allows for a more accurate calculation of the economic burden at the population level. Third, the use of a bottom–up methodology based on empirical data from surveys and administrative records strengthens the validity of the estimates by minimizing generalized extrapolations. Additionally, the study has covered almost the entire pediatric population with CP in the region, reducing selection bias and improving the generalizability of the results. The access to a representative population-based dataset from the epidemiological registry of Navarra further reinforces the robustness of the findings. Furthermore, the inclusion of the Zarit Scale to assess the emotional and social burden of caregivers allows for a more holistic evaluation beyond financial costs. The study also provides a detailed estimation of indirect costs, particularly the loss of caregiver productivity, highlighting an often-underestimated financial burden that is crucial for policy planning and resource allocation (3). Finally, this is the first study to comprehensively assess CP-related societal costs in Spain, filling an important gap in the literature.

### 4.1 Limitations

This study has several limitations. Accurately capturing the costs associated with CP was challenging due to the unique characteristics of this population (12, 18). Heterogeneity in the types and severity levels of CP introduces variability in resource utilization patterns and associated costs (9, 14). In addition, certain expenditures, such as transportation costs, home modifications, and hours of care, may be underestimated due to reliance on family reports. Nevertheless, the estimate of 9.5 h of care per day is consistent with existing literature and falls within the expected range of care demands (30, 43). Another important limitation is the exclusion of the patient's loss of productivity, given the complexity of disability-related work limitations because the patient with CP has a low capacity to work and tends to become severely disabled in middle age. This has a negative impact on labor productivity and, in the absence of significant public subsidies, household income will undoubtedly decrease significantly. In addition, the reduction in income will influence the affordability of medical care and support services for the CP patient. Therefore, assessment of this component would be essential for future research on the wider economic impact of CP. This study only estimated costs over a 1-year period without extrapolating to longer time horizons. However, we consider this year to be representative, as there were no extraordinary events that would have significantly altered the usual costs associated with the disease. Finally, a key limitation is the regional scope of the study. However, the region is representative in terms of health and social support policies, being comparable to other Spanish regions, although differences in resource allocation may limit national generalisability. Future research should be extended to multiple regions to refine cost estimates and policy recommendations.

### 4.2 Policy roadmap

Based on our findings, particularly the high out-of-pocket share borne by families and the disproportionate burden on those with more severe motor impairments, we propose a structured policy roadmap to guide national action. In the *short term*, policy should prioritize the expansion of respite-care funding and the creation of means-tested subsidies for essential therapies and assistive devices. These interventions should be stratified by GMFCS level and socioeconomic status to target those with the greatest unmet needs. In the *medium term*, a national CP registry should be established to support planning and equity in service provision. Simultaneously, multidisciplinary early-intervention hubs should be scaled across regions to ensure timely and coordinated support during the most critical developmental periods. In the *long term*, Spain should work toward implementing an integrated, value-based care pathway for individuals with CP across the lifespan. Additionally, social-security reforms that credit the unpaid care work of primary caregivers would promote long-term economic equity. This roadmap aligns with international policy efforts and reflects the urgency and scope of the economic burden revealed in this study.

## 5 Conclusions

The economic burden of pediatric CP in Spain is substantial and primarily borne by families, underscoring the need for improved public policies. Drawing from international best practices, Spain could enhance its support framework by expanding respite care, increasing public funding for essential therapies, and providing targeted financial aid to families. Public policies should ensure the funding of complementary therapies, particularly for low-income families, to improve functional outcomes and the wellbeing of children with CP. Future research should focus on the cost-effectiveness of these interventions, ensuring that resource allocation maximizes both economic efficiency and quality of life for affected individuals.

## Data Availability

The data from this study are available upon justified request through the corresponding author to the institution that holds the data (University Hospital of Navarra).

## Supplementary material

- Questionnaire to measure the economic burden of cerebral palsy (EBQ-CP). Own elaboration for the study.
- Supplementary Figure S1. Flowchart of patient selection and inclusion process in the study population with pediatric cerebral palsy.
