# Supplementary material for: The economic and social burden of pediatric cerebral palsy in Spain: a cost-of-illness study
Source: Front Public Health. 2025 Jul 23;13:1589114. doi: 10.3389/fpubh.2025.1589114 (PMC12325214; doi:10.3389/fpubh.2025.1589114)
Supplement: Supplementary file 1 [file Data_Sheet_1.pdf]

Economic Burden Questionnaire of Cerebral Palsy (EBQ-CP)

Includes: A Diary Informal care monitoring module to calculate productivity loss
And Impact of care and financial situation: caregiver wellbeing section

QUESTIONNAIRE FOR PRIMARY CAREGIVERS*

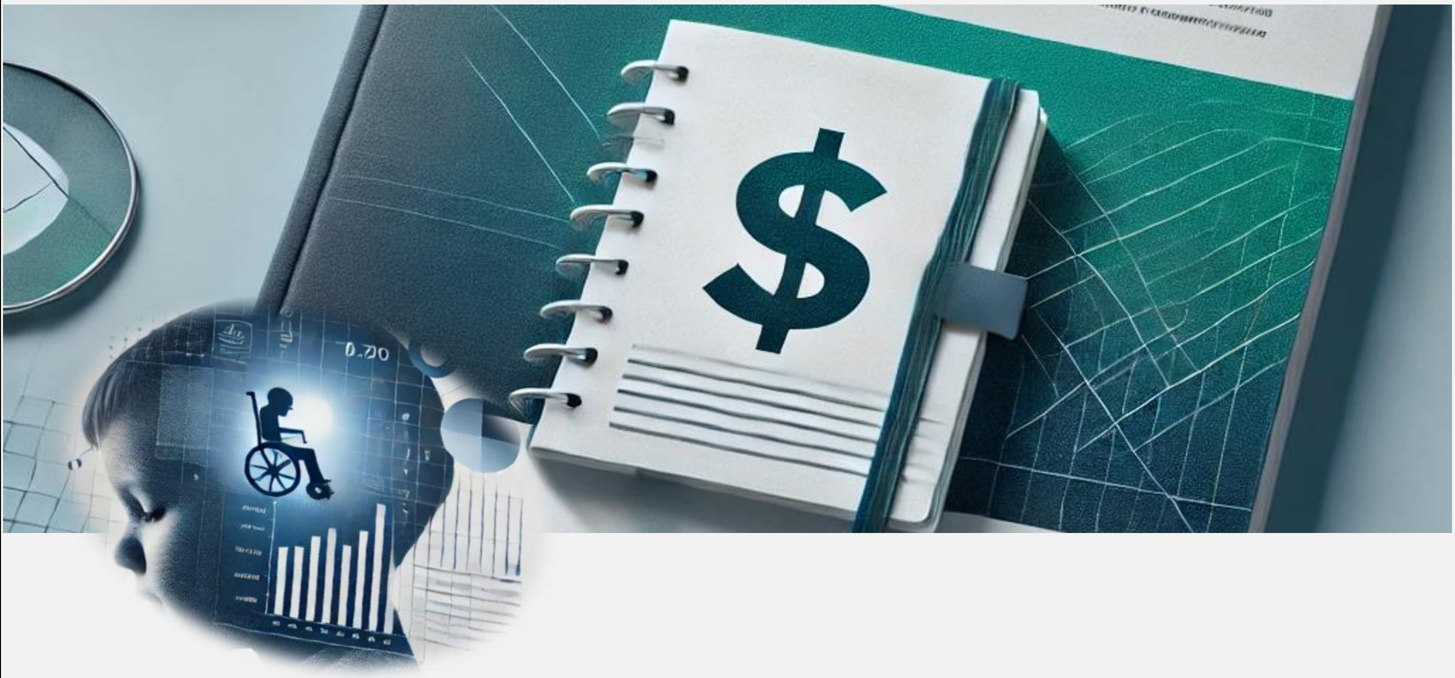

**Applicable version: The sections on medical costs and clinical characteristics of the patient should be completed with the help of the referral neuropediatricians. It is advised that all public health care cost data can be cross-checked with administrative data.*

APPLICATION MANUAL-USER GUIDE

INFORMATION FOR THE INTERVIEWER AND THE RESEARCHER

The *Economic Burden Questionnaire of Cerebral Palsy (EBQ-CP)* is designed to estimate the Cost of Illness (COI), specifically from the perspective of families. It was developed to be answered by the primary caregiver and its data should be supplemented or verified by administrative and clinical records of the patient. The questionnaire is structured in three main sections:

A. SOCIODEMOGRAPHIC AND SOCIO-ECONOMIC CHARACTERISTICS: This section contains information regarding the main caregiver(s) in the aspects mentioned that allow the main characteristics of the caregiver and his/her situation to be identified. This part is intended to be administered as a semi-structured interview by the interviewer.

B. DIRECT ECONOMIC BURDEN OF CEREBRAL PALSY: The direct economic burden of the disease refers to those costs that can be health or non-health related and that are closely related to the disease and its development in the patient. This section is subdivided into two parts:

1) Direct healthcare costs reported by the primary caregiver and corroborated with administrative data:

This section captures expenses related to the patient's medical care, initially referring to *specialist consultations and required diagnostic tests*. It also includes all *costs associated* with various *types of treatments: conventional, related to comorbidities, complementary*, and *alternative therapies*. In addition, *hospitalization costs* are considered. This part of the questionnaire is designed to be administered through a semi-structured interview conducted by the interviewer with the primary caregiver, and the responses can subsequently be cross-checked with data from administrative records and the patient's clinical history.

2) Direct non-healthcare costs reported by the primary caregiver:

This section includes *non-medical expenses* incurred by the family due to cerebral palsy. It covers: acquisition of *special equipment for rehabilitation* or other purposes; *home and vehicle modifications* related to CP; *nutritional or special dietary products* for the patient; *education, training*, and integration-related expenses; *formal and informal care paid* by the family; *adapted clothing and personal items*; legal advocacy and protection; and economic protection or planning. This part of the questionnaire is also intended to be administered through a semi-structured interview with the caregiver, although it can also be self-completed by the caregiver if necessary.

C) INDIRECT ECONOMIC BURDEN OF CEREBRAL PALSY: Indirect costs primarily *refer to productivity losses* experienced by both the person with cerebral palsy and their primary caregiver. In this context, the *focus is placed on the caregiver's loss of productivity*. This final section of the instrument is divided into two components:

1. **General productivity loss associated with caregiving for a person with CP:**

   This section assesses the overall loss of productivity related to caregiving duties, *using broader time units (e.g., weeks or months)* to capture the *long-term impact of caring for a person with cerebral palsy*. It is designed to be administered through a semi-structured interview conducted by an interviewer with the primary caregiver. However, it may also be self-completed by the caregiver if an interviewer is not available.

2. **Daily productivity loss and well-being associated with caregiving for a person with CP:**

   This component is structured as a *caregiver diary*, intended to be completed by the caregiver on a *daily basis*. It records the number of hours dedicated to each *caregiving activity performed for the person with CP*. The objective is for the caregiver to complete this diary over the course of one month and subsequently submit it to the health economics evaluation team.

BRIEF PRESENTATION TO BE MADE BY THE INTERVIEWER TO THE CAREGIVER

Interviewer Introduction:

Good morning/afternoon. My name is (name), and I am representing (institution name). We are conducting a study to better understand the costs that families face when accessing healthcare services and providing daily care to individuals with cerebral palsy (CP) throughout different stages of life. Our goal is to assess the economic impact of CP on households.

It is important for you to know that your participation in this study is entirely voluntary. We would greatly appreciate your willingness to take part, but you are completely free to decline. If you choose not to participate, there will be no consequences for you, and it will not affect the medical care or support you receive at this facility. You will not lose any rights or benefits to which you are entitled.

If you decide to participate, please note that you may withdraw from the study at any time without providing a reason. All the information you share with us will be kept strictly confidential. At some point during the interview, we may ask about your personal and household income. We assure you that this information will not be shared with any tax or social service authorities, and it will remain confidential even after the study concludes.

This survey will take approximately 60 minutes to complete.

Do you have any questions?

Would you like to participate? Yes / No
If yes: **Thank you!**
If no: **May we ask why?**

1. Insufficient language proficiency
2. Lack of time
3. Discomfort or unwillingness
4. Unspecified reason

BEGIN THE SEMI-STRUCTURED INTERVIEW

INFORMATION TO BE PROVIDED BY THE INTERVIEWER IN EACH SECTION TO THE PRIMARY CAREGIVER (RESPONDENT)

0. IDENTIFIER OF PRIMARY CAREGIVER(S)

Data on the care situation

We are now going to begin the questionnaire: Objective Burden – Economic Burden of Cerebral Palsy (EB-CP). To start, we will ask you to answer *seven initial questions* that will allow us to identify you as the caregiver of one of the children participating in this study. Next, we will ask about the *number of individuals involved in the care of the patient*, and based on your specific situation, you will be assigned a *caregiver type*. The *type of interview* will also be defined, and finally, we will request *contact information* in case it becomes necessary to gather any missing data or to reschedule the interview if it cannot be completed today.

A. SOCIO-DEMOGRAPHIC AND SOCIO-ECONOMIC CHARACTERISTICS

Data on Primary Caregivers

In this section, you will be **asked 13 personal questions** related to your role as the primary caregiver of a child with cerebral palsy (CP). Please listen carefully to the questions asked by the interviewer**. They will cover topics such as your gender, age, marital status, nationality, educational level, employment status, economic situation, family circumstances, and access to health services**. If at any point you have questions or need clarification, feel free to ask. Please remember that all your responses will be completely anonymous, no one will be able to identify who provided the answers. We encourage you to respond openly and honestly, as your answers will have no negative consequences. The information gathered will help us better understand the reality of caregivers of children with CP, raise awareness about your role, and provide real data to support policies and services that meet both your needs and those of the child

B. DIRECT ECONOMIC BURDEN

Module: Direct Health Costs Reported by the Primary Caregiver and Neuropediatricians

Explain to the caregiver that the purpose of this section is to collect detailed information about direct medical costs related to the care of a child with cerebral palsy (CP), based on data provided by the primary caregiver and, when applicable, verified with neuropediatricians and clinical records. All questions refer to the past 12 months.

SECTION 1. MEDICAL CARE: GENERAL COSTS OF CEREBRAL PALSY

Objective: To gather data on the general medical care costs directly related to CP, including outpatient consultations, diagnostic tests, and hospital admissions.

Instructions for the Interviewer:

1. Ask the caregiver to recall and report all medical services the child received in the last 12 months, related to CP or its comorbidities.
2. If the caregiver is unsure, indicate that some data will be verified with their healthcare provider.
3. For each cost item (consultation, transport, medication, etc.), ask for: Number of visits or tests, Average time per visit, Transportation method (own/public), Other associated costs (parking, food, etc.)
4. Encourage the caregiver to consult receipts, appointment records, or calendars if needed.

Table A. GENERAL OUTPATIENT CONSULTATIONS AND TEST

Record visits to general practitioners and specialists (e.g., neuropediatricians, rehabilitators, ophthalmologists). Include: *Number of visits, Time spent per visit, Time taken to travel, Mode of transport, Transport costs*

Diagnostic tests**,** ask about specific tests conducted in the past year (e.g., MRIs, X-rays, EEGs). For each*: Record the number of times, Estimate the duration of each test, Document travel and related costs*

Table B. HOSPITALIZATION

Ask if the child was hospitalized in the last 12 months due to CP or its complications:

- If No, continue to the next section.
- If Yes, complete the hospitalization table with: Name of the hospital, Type of admission (emergency, >48h, ICU), Duration of stay, Caregiver time accompanying the child, Transportation and accommodation costs, Other out-of-pocket costs (e.g., phone, food)

SECTION 2. CONVENTIONAL TREATMENTS

(These are standard medical treatments provided by the public health system.)

Objective: To record costs related to medical treatments regularly prescribed for CP and received within the last 12 months.

Instructions:

1. Ask the caregiver about each type of treatment: motor therapies, pharmacological treatments, surgeries, or psychosocial support.
2. Complete the table for each service used: Number of visits, Average duration, Transportation details and costs, Out-of-pocket expenses on medications (prescribed and non-prescribed), Other expenses (e.g., parking, meals)

SECTION 2.1. TREATMENTS FOR COMORBIDITIES

(Conditions frequently associated with CP such as epilepsy, nutritional, gastrointestinal or sensory disorders.)

Instructions:

1. Ask if the child is receiving any treatment for comorbidities. If No, move to the next section.
2. If Yes, fill in: Type of comorbidity (e.g., epilepsy, cognitive deficit, psychiatric conditions), Type of treatment, Number of visits, Duration and travel details, Medication costs, Other expenses

SECTION 3. COMPLEMENTARY TREATMENTS

(These are non-public treatments that are used alongside conventional therapies and supported by clinical evidence.)

Instructions:

1. Ask: "Has your child received any complementary treatments in the past 12 months for CP or related conditions?"
2. If Yes, complete: Type of treatment (e.g., occupational therapy, sensory integration, cognitive therapy), Location (private or mixed public-private centre), Number and duration of visits, Travel and other related expenses, Out-of-pocket payment for the treatment.

SECTION 3.1. ALTERNATIVE TREATMENTS

(These are treatments that replace conventional or complementary ones, are not covered by the public system, and are usually based on controversial or unproven efficacy.)

Instructions:

1. Ask: "Has your child received any alternative treatments (e.g., homeopathy, acupuncture) in the last 12 months?"
2. If Yes, complete: Type of treatment (e.g., hippotherapy, osteopathy, vision therapy), Number of sessions, Average duration, Transport Method, Treatment cost and additional out-of-pocket costs

RECOMMENDATIONS FOR INTERVIEWERS

- Be patient and supportive. Some caregivers may feel overwhelmed.
- Use a conversational tone and allow time for reflection.
- Help the caregiver estimate missing data if necessary (e.g., "Would you say it was about once a week, once a month...?").
- Write "Not known" or "Not applicable" when data cannot be obtained.
- Always remind the caregiver that some responses will be validated with the clinical team if needed.

Direct Non-Health Costs reported by the primary or family caregiver

Explain to the main caregiver of the patient that in this section, we are going to collect the expenses that are assumed by the main caregiver or a family member directly related to the care of the patient, but that do not correspond to medical services or health treatments.

SECTION 4. SPECIAL EQUIPMENT AND AIDS FOR MOBILITY AND COMMUNICATION

Objective: To identify costs related to the purchase, maintenance, or replacement of special equipment or assistive technologies required due to the child's CP.

Instructions:

1. Ask: "Because of your child's condition, have you had to buy any special or rehabilitation equipment (e.g., wheelchairs, crutches, communicators) in the last 12 months?"
2. If Yes, complete the table recording: *Type and purpose of the equipment, Purchase price and Quantity, Duration of use, Funding source (e.g., insurance, government program, out-of-pocket), Repair and maintenance costs, Replacement frequency, Additional associated costs, any access difficulties (e.g., long waiting lists, bureaucratic barriers)*

SECTION 5. MODIFICATIONS TO THE HOME AND/OR VEHICLES

Objective: To document structural adaptations to the child's living environment made due to the disease.

Instructions:

1. Ask: "Have you had to make any modifications to your home or vehicle because of your child's condition?"
2. If Yes, complete the table with details for each modification made in the last 5 years, including: *Type of modification and the reason it was needed, Estimated cost and out-of-pocket expenses, Funding Source, Number of times such modifications have been required, Date or year of the intervention*

SECTION 6. CLOTHING, NUTRITION, AND PERSONAL CARE

Objective: To assess expenses on items needed for basic care due to CP or related comorbidities.

Instructions:

1. Ask: "In the last 12 months, have you purchased any of the following for your child? Incontinence products (e.g., diapers), nutritional supplements, or special clothing or shoes?"
2. If Yes, record relevant information on product type, frequency of purchase, total costs, and any difficulties in accessing these products.

SECTION 7. EDUCATION, TRAINING, AND INTEGRATION

Objective: To gather data on educational services and related expenses.

Instructions:

1. Ask: "Does your child currently attend a day care center, kindergarten, school, or another educational center?"
2. If Yes, ask whether the institution is public, private, or concerted, and complete the table with: *Name and type of institution, Daily attendance Schedule, Presence of a dedicated caretaker at the center, Time invested by the caregiver (transportation and waiting), Tuition fees, Additional costs (e.g., canteen, excursions, school supplies), Transportation costs*

Note: These data will be cross-verified with education records where available.

SECTION 8. LEISURE, RESPITE CARE, AND HOLIDAYS

Objective: To evaluate indirect costs and caregiver support through leisure, holidays, or formal care services.

Instructions:

1. Ask the caregiver: "Have you paid for a formal caregiver or participated in any leisure or holiday activities with the child in the past 12 months?"
2. If Yes, complete the table indicating: *Type of service or activity (formal care, leisure, holidays), Time spent and frequency, Unit and total annual cost, Funding source, Any comments or notes (e.g., access difficulties, caregiver burden)*

Note: Respite care refers to any formal arrangement intended to give primary caregivers temporary relief.

SECTION 9. DISABILITY ALLOWANCE

Objective: To assess financial support received by the child and the primary caregiver.

Instructions:

1. Ask: "Has the child received any disability-related funding or allowance in the past 12 months?"
2. If Yes, indicate the total amount received using pre-defined income brackets or the exact amount if known.
3. Then ask: "Has the main caregiver received any financial subsidy for care (e.g., CUME) in the past 12 months?
4. If Yes, report the estimated range or the exact amount received.

SECTION 10. OTHER OUT-OF-POCKET EXPENSES

Objective: To capture any remaining costs not yet reported.

Instructions:

1. Ask:
   - "Do you pay for a private health or life insurance for your child?"
   - If YES, record the estimated monthly payment range.
2. Ask:
   - "Are there any other significant expenses not yet mentioned?"
   - If YES, specify the type and total cost incurred in the last 12 months.

Finally, ask: "Of all the out-of-pocket expenses mentioned in this questionnaire, which category had the greatest financial impact on your household?" Let the caregiver choose from a predefined list (e.g., treatments, mobility aids, home modifications, etc.).

C. INDIRECT ECONOMIC BURDEN

Section C, on Direct Economic Burden, is divided into two sections. The first contains four general questions on caregiving, which will be explained below. The second includes twelve questions; the first is in the form of a diary or detailed record of the caregiver's daily hours spent with the patient with cerebral palsy. The remaining ten questions focus on the impact of caregiving and the financial situation, assessing the caregiver's well-being.

Section 11. Loss of Productivity associated with disease care

In the first section you will be asked questions about how long you have been providing care to the child with CP, in a general time range of days, weeks and years. Then you will be asked about the main activities in which you provide care in general in your work as a primary caregiver. (Go to questions 1,2,3,4)

Diary of hours of care per day

1. In the second part, the caregiver will be asked to keep a daily diary for 4 consecutive weeks, recording the hours spent each day on the different care or assistance activities performed for the patient. A specially designed form will be provided for them to fill out comfortably at home.

It is important that the time is recorded as accurately as possible, since these records will later be reviewed and adjusted with appropriate weights according to the type of care activity carried out, based on established dependency assessment criteria.

(Give or send the diary sheet to the main caregiver and/or family member)

Questions 2-12 Impact of Caregiving and Financial Situation

Most demanding task: Ask the interviewee to identify the task that has affected their life the most. There is no need to justify their choice.

Impact on physical, mental, and social health: Inquire if they have noticed any deterioration in these areas over the past year due to caregiving. Then, ask them to rate it from 1 to 10.

Treatment received: Ask if they have received medical or psychological care in the last 12 months to cope with the caregiving burden. Record the type of treatment and whether it was public or private.

Treatment cost: Place the expense within one of the provided ranges, even if it is an estimate.

Perception of Financial Situation: The following questions are subjective assessments. Explain that they should answer based on their personal experience. Present the options clearly, then ask them to indicate a number from 1 to 10, where 1 represents the worst situation or highest stress, and 10 the best situation or least concern. Take these questions calmly. Some deal with sensitive topics like stress, savings, or emergencies. Be empathetic if they show discomfort.

At the end of the interview: Thank them for their time and honesty.

Remind them that their experience as a caregiver is very valuable for better understanding the needs of families affected by cerebral palsy. If the activity diary was given, agree on how and when to return it or follow up.

Glossary of Key Terms – EBQ-CP Questionnaire

This glossary is intended to facilitate the understanding of key terms used in the *Economic Burden Questionnaire of Cerebral Palsy (EBQ-CP)*. Definitions follow international standards in economic evaluation, health services research, and caregiving studies.

Types of Treatment

- **Conventional Treatment**
  Medical care that is part of standard evidence-based clinical practice and covered by the public health system. Includes physical therapy, speech therapy, botulinum toxin injections, surgeries, and pharmacological management.

- **Complementary Treatment**
  Interventions used **together with conventional treatments**, typically not full included in the public healthcare system. These treatments are intended to support functional outcomes and are paid out-of-pocket by families.
  Examples: Intensive physiotherapy, intensive speech therapy and intensive occupational therapy

- **Alternative Treatment**
  Interventions used **instead of conventional or complementary care**, often without robust scientific evidence. Typically provided in private settings and fully paid by families.
  Examples: Hippotherapy, acupuncture, homeopathy, Therasuit method.

Types of Caregivers

- **Informal Caregiver (Type 1)**
  A **non-paid family member** who provides care for the person with CP (e.g., mother, father, grandparent).

- **Informal Caregiver (Type 2)**
  A **paid family member** who receives compensation for providing care, usually through public subsidies or informal arrangements.

- **Formal Caregiver (Type 1)**
  A **non-relative hired by the family** to assist with daily care tasks.

- **Formal Caregiver (Type 2)**
  A **healthcare professional assigned by the public health or social care system** to support the family.

- **Primary Caregiver**
  The person who spends the most time caring for the child with CP and is most familiar with their health and daily needs.

Type of Costs

- **Direct Health Costs**
  Expenses directly related to the child’s medical care, such as consultations, medications, therapies, hospitalizations, and diagnostic tests.

- **Direct Non-Health Costs**
  Non-medical expenses due to CP, including transport, special equipment, home modifications, special clothing, and school-related fees.

- **Indirect Costs**
  Productivity losses or missed work opportunities by the caregiver, often measured through time dedicated to care.

- **Out-of-pocket Expenses**
  Any costs paid directly by the family that are **not reimbursed** by public or private insurance schemes.

Glossary of Key Terms – EBQ-CP Questionnaire

Measurement and Valuation Methods

- **Proxy Good Method**
  An approach used to estimate the **monetary value of unpaid informal care**, by assigning a market wage equivalent to the hours of care provided (e.g., the cost of hiring a professional caregiver).

- **Co-production Bias**
  A bias that may occur when time spent on care is overestimated because multiple caregiving activities happen simultaneously. This is why reported hours are capped at 16 hours per day.

- **Care Diary**
  A structured tool for recording daily time spent on different caregiving tasks. Used to assess informal care burden over a defined time period (typically 4 weeks in this instrument).

Social and Financial Support

- **Disability Allowance**
  Public financial support granted to children with disabilities or their families, intended to offset some of the additional costs of care.

- **CUME (Caregiver Subsidy)**
  A Spanish-specific public benefit for caregivers, often granted to family members providing extensive daily care.

- **Health or Life Insurance**
  Voluntary private coverage purchased by the family. This may include insurance for rehabilitation services or life insurance linked to chronic care planning.

Educational and Social Services

- **Special School / Center**
  Institutions designed specifically for children with disabilities, offering tailored educational support and often additional therapies.

- **Normal School with Support**
  Regular education setting that provides additional individualized support to accommodate a child with CP.

- **Respite Care**
  Temporary formal care provided by a third party to allow the primary caregiver rest. Can include short stays in institutions or professional caregivers at home.

Functional Classifications

- **GMFCS (Gross Motor Function Classification System)**
  A standardized tool for classifying the severity of motor impairment in children with CP from Level I (least severe) to Level V (most severe, full dependence).

Time Frames Used in the Questionnaire

- **Last 12 months**
  Default period for cost-related questions, unless otherwise specified.

- **Last 5 years**
  Applied only to home and vehicle modification expenses, due to their infrequent nature

Start the interview from the questionnaire below

0. IDENTIFIER OF PRIMARY CAREGIVER(S)

Identifier of primary caregiver(s)

The following questionnaire is addressed to the primary caregiver of:

Name _______________

*Sex*_______________

*Age*_______________

Postcode Place of residence ____________________

0.a. Is there more than one main caregiver taking care of the patient?

Yes ☐ *No* ☐

If there is more than one caregiver, please indicate how many:

A. 2 ☐

B. 3 ☐

0.b. Type of caregiver

Indicate if you are a caregiver:

A. *Informal type 1 (Unpaid Family Member)* ☐
B. *Informal type 2 (Paid family member)* ☐
C. *Formal Type 1 (Non-relative and paid by the patient's family)* ☐
D. *Formal type 2 (Health professional who was assigned by the public services)* ☐

PRIMARY CAREGIVER: The caregiver who spends the most hours caring for the patient and therefore is assumed to know the patient best.

0.c. **Is the respondent to this questionnaire the main caregiver of the person with a disability (3 years and older) or of the child with a disability (2 to 5 years) identified in this section?**

Yes ➡ *Go to 0.e*

No ➡ *Go to 0.d*

0.d. Reason for proxy information:

Prolonged absence Caregiver only at night Lack of knowledge of language More than one caregiver Other ☐ ☐ ☐ ☐ ☐

0. e. Type of interview:

In person ☐

Phone ____________________ ☐

A. SOCIODEMOGRAPHIC, SOCIOECONOMIC AND CLINICAL CHARACTERISTICS

Data on Primary Caregivers

***0.* Gender**

- ☐ Male
- ☐ Female

***1.* Age**

☐ years

☐ Don't know / No answer

***2.* What is your marital status?**

Single ☐
Married/free union ☐
Widowed ☐
Divorced ☐
Separated ☐
Unmarried partner ☐ ☐

***3.* What is your nationality?**

Spanish ☐

Foreign ☐ ____________________

Spanish and foreign ☐ ____________________

***4.* What is your highest completed level of education you achieved?**

- ☐ No education, cannot read and write
- ☐ Primary Education
- ☐ Secondary Education
- ☐ Vocational Training
- ☐ Tertiary Education
- ☐ None of the above

5. What is your main situation in relation to the labour market?

- ☐ Working full or part-time
- ☐ Unemployed (i.e. out of work and looking for a job)
- ☐ Retired, early retired, retired from previous economic activity Studying
- ☐ Unable to work
- ☐ Engaged in household chores and/or care work
- ☐ He has left the labour market to care for a patient with CP.
- ☐ Don't know / No answer

6. Do you work part-time? If the answer is No, go to question 7.

Yes ☐ No ☐

6.1. How many hours do you work per week?

A. Less than 10 hours ☐
B. Between 10 and 20 hours ☐
C. Between 20 and 30 hours ☐
D. Between 30-40 hours ☐

6.2. You work only part-time because the other part of your time is mainly spent on care OR there is another reason. If yes, please specify:

Yes ☐ No ☐ Other reason ____________________

7. If the main caregiver is a relative of the patient, please answer the following question: Who is the main income provider(s) in the household? Please tick all that apply:

1. Wife/Mother ☐

2. Husband/father ☐

3. Siblings/Children ☐

4. Extended family (grandparents, etc.) ☐

5. The person affected by CP* ☐

6. Other (please specify) ☐

*Disability allowance

8. If the main caregiver is a relative of the patient, please answer the following question: What is the average yearly household income?

1) Less or up 30.000€ ☐
2) 31.000€ - 52.000€ ☐
3) 53.000€ - 72.000 € ☐
4) More than 73.000€ ☐
5) Did not wish to disclose ☐

9. If the caregiver is a relative of the patient and is not currently working, please answer the following question: Is the reason for not working outside the home related to your relative's CP?

Yes ☐ No ☐

10. If the caregiver is a relative of the patient and is not currently working, please answer the following question:
When was the last time you were working?

(mm--------/yy---------)

11. As a primary caregiver, do you usually have help from others to carry out the patient's care?

Yes ☐ No ☐

12. If the answer is yes, please indicated whether:

A. Receives help from another family member ☐
B. Receives help from an external person hired by the family to care for the patient. ☐

13. How long does it take to reach the nearest hospital or rehabilitation center from your place of residence?

A. Easy access: Less than 30 minutes ☐
B. Limited access: Between 30 and 90 minutes ☐
C. No local access: More than 90 minutes ☐

General clinical data of pediatric patients*

What is the type of CP of the patient?

☐ Spastic (Muscles appear stiff and tight. From motor Cortex damage)
☐ Ataxic (Characterised by shaky movements. Affects balance and sense of positioning in space. From Cerebellum damage)
☐ Dyskinetic (Characterised by involuntary movements such as dystonia, athetosis and/or chorea. Damage to the Basal Ganglia)
☐ Mixed
☐ I don't know

Please choose the most appropriate level of severity:
For more information please refer to GMFCS (Gross Motor Functional Classification System)

☐ Level I - I walk without limitations
☐ Level II - I walk with limitations
☐ Level III - I walk using a hand-held mobility device
☐ Level IV - I move around with limitations. I may use a powered mobility.
☐ Level V - I am transported in a wheelchair
☐ I don't know

Please tick for any other associated impairments and other conditions

☐ Epilepsy
☐ Intellectual
☐ Visual
☐ Hearing
☐ Speech
☐ ADHD
☐ ASD
☐ None
☐ Other: ____________________________
(Select as many as applicable)

** Section for the neuropediatricians*

B. DIRECT ECONOMIC BURDEN

Direct Health Costs reported by the main Caregiver and Neuropediatricians

1. MEDICAL CARE: GENERAL COSTS OF CEREBRAL PALSY

i. Fill in the table below and record the applicable general cost of CP for the patient that have occurred in the last 12 months:

TABLE A

GENERAL COSTS RELATED TO CEREBRAL PALSY IN THE LAST 12 MONTHS						
HOSPITAL/MEDICAL CENTRE VISITS	Time spent by the caregiver to attend the consultation		TRANSPORT COSTS			TOTAL
			Time taken to travel	Means of transport		
CONCEPT	No. of visits	Average time	Average time	Own	Public	
General practitioner						
CONSULTATIONS WITH SPECIALISTS						
Neonatology						
Neuropediatric						
Traumatology						
Rehabilitation						
Ophthalmology						
Digestive Nutrition						
Otorhinolaryngology						
Pneumology						
Endocrinology						
Paediatric surgery						
Chronic Pathology Unit						
TESTS	No. of tests					
RM						
Tomography Computerized						
Ultrasounds						
X-rays						
Electroencephalogram						
Swallowing test						
Ph-metrics						
Electrophysiology						
Other evidence additional						
Sleep study						
Blood test						
Other questions neurophysiological						

HOSPITALISATION

Data to be checked with the referring neuropediatricians/GP and administrative records.

i. In addition to the treatments received by the patient with CP, has he/she been hospitalized for the disease or its comorbidities?

Yes ☐ No ☐

If the answer is yes, please fill in the following table where data on hospitalisation for the last 12 months will be recorded:

TABLE B

Hospitalisation costs by type

Name of the Hospital o Health Care Center	Type of hospitalisation			Time spent by the caregiver accompanying the patient	TRANSPORT EXPENDITURE			Accommodation costs	Other out-of-pocket expenses (phone calls, parking, food, taxes, etc.)	Total costs during hospitalisation
	Emergency Day Hospital -48hours	Admission ≥48 hours	UCI +48 hours		Time taken to travel	Means of transport				
	Average time (Hours-days)	Average time (Hours-days)	Average time (Hours-days)	No. of hours on average	No. of hours on average	Own	Public			

* If it was a private clinic or hospital, specify this in other out-of-pocket expenses

TREATMENTS

2. CONVENTIONAL TREATMENTS

Data to be checked with the referring neuropediatricians/GP and administrative records.

Treatment that is included in standard medical practice or within the public health system to treat CP.

i. Please fill in the table below on the costs of conventional treatments or treatments carried out in hospital that have taken place in the last 12 months for the patient with CP:

COSTS RELATED TO CONVENTIONAL CP TREATMENT LAST 12 MONTHS									
**HOSPITAL VISITS**	**Time spent by the caregiver to attend the consultation**		**TRANSPORT EXPENDITURE**			**Out-of-pocket Expenditure on Medicines in EUR**		**Other out-of-pocket expenses (Calls, car parks, food, Fees etc)**	**Total costs during treatment**
			**Time taken to travel**	**Means of transport**					
**Type of treatment**	**No. of visits**	**Average time**	**Average time**	**Own**	**Public**	**Non-prescribed**	**Prescribed**		
**Motor Interventions: Therapies and Rehabilitation**									
Occupational therapy									
Motor physiotherapy									
Respiratory physiotherapy									
Speech therapy									
**Tone and pain management**									
Botulinum toxin									
Oral baclofen									
Benzodiazepines									
Analgesics (Paracetamol, Ibuprofen, metamizol									
Other									
**Psychosocial Interventions**									
Social Work									
Neuropsychology									
**Surgical**									
Surgery for dislocated hip									
Multilevel surgery									
Tracheostomy									
Gastrostomy									
Anti-reflux surgery									
Neurosurgery									
ENT surgery									
Corrective surgery of scoliosis									
Others									

TREATMENTS FOR COMORBIDITIES

Data to be checked with the referring neuropediatricians/GP and administrative records.

Comorbidity: Refers to related disorders or diseases that occur in the same person and are often associated, in this case with CP.

ii. **Is the patient receiving other treatments to treat co-morbidities?**

Yes ☐ No ☐

If the answer is yes, please fill in the following table where this type of treatment will be recorded for the last 12 months:

COSTS RELATED TO THE TREATMENT OF CP COMORBIDITIES LAST 12 MONTHS									
HOSPITAL/HEALTH CENTRE OR OTHER FACILITIES	Time spent by the caregiver		TRANSPORT EXPENDITURE			Out-of-pocket expenditure on medicines		Other out-of-pocket expenses (Calls, parking, food, Fees etc.)	Total costs during the treatment
			Time taken to travel	Means of transport					
Type of comorbidity	No. of times	Average time	Average time	Own	Public	Prescribed	Non-prescribed		
Epilepsy (Anti-crisis drugs/Non-pharmacological therapies: ketogenic diet and stimulator) of the vagus nerve)									
Cognitive deficit (Social support)									
Psychological and Psychiatric disorders (Psychopharmaceuticals).									
Nutritional disorders (Milk, vitamin supplements, vitamins, etc.)									
Gastrointestinal disorders (Drugs, Laxatives, Thickeners)									
Neurosensory disorders: Vision Hearing (Hearing aids, glasses, etc.)									
Medication Treatment Non-Invasive Ventilation									
OTHER SPECIFIC									

3. COMPLEMENTARY TREATMENTS

COMPLEMENTARY TREATMENT: Complementary to treatments included in standard medical practice to treat CP, they are not included, but are associated with proven and effective treatments.

i. Is the patient receiving any complementary treatment for CP or its comorbidities?

Yes ☐ No ☐

If the answer is yes, please fill in the following table where this type of treatment will be recorded for the last 12 months:

COSTS RELATED TO COMPLEMENTARY TREATMENT OF CP LAST 12 MONTHS									
VISITS TO PRIVATE CENTRES PUBLIC-PRIVATE	Time spent by the caregiver to attend the consultation		TRANSPORT EXPENDITURE			Out-of-pocket treatment costs in EUR		Other out-of-pocket expenses (Calls, car parks, food, Fees etc)	Total costs during treatment
			Time taken to travel	Means of transport					
Type of treatment	No. of visits	Average time	Average time	Own	Public				
REHABILITATION									
Occupational therapy									
Motor physiotherapy									
Respiratory physiotherapy									
Speech therapy									
Paediatric exoskeleton									
Sensory integration									
Other									
Cognitive Interventions									
Cognitive stimulation									
Psychology									
Surgical									
Surgery for dislocated hip									
Public hospital referral									
External surgeries									
Private hospital consultations									
OTHER SPECIFIC									

ALTERNATIVE TREATMENTS

ALTERNATIVE TREATMENTS: These are intended to replace conventional and complementary treatments. In practice, they are treatments that are not covered by the health system and are offered by private centres. Their efficacy must be evaluated based on scientific evidence, their validity for the diseases they claim to treat and their safety.

ii. Does the patient receive alternative treatments for CP or its co-morbidities?

Yes ☐ No ☐

If the answer is yes, please fill in the following table where this type of treatment will be recorded for the last 12 months:

COSTS RELATED TO ALTERNATIVE TREATMENT OF CP LAST 12 MONTHS									
VISITS TO PRIVATE CENTRES	Time spent by the caregiver to attend the service		TRANSPORT EXPENDITURE			Out-of-pocket treatment costs in EUR		Other out-of-pocket expenses (Calls, car parks, food, Fees etc)	Total costs during treatment
			Time taken to travel	Means of transport					
Type of treatment	No. of times	Average time	Average time	Own	Public				
ALTERNATIVE THERAPIES									
Hippotherapy									
Hormone treatment growth									
Osteopathy									
Acupuncture									
Hyperbaric oxygen therapy									
Peto Method									
Homeopathy									
Vision Therapy (Optometrist)									
Therasuit									
OTHER SPECIFIC									

Direct Non-Health Costs reported by the primary caregiver or a family member*

*In case the main caregiver is not a family member, as in this type of information the family is the main source for the type of expenditure.

4. SPECIAL EQUIPMENT AND AIDS FOR MOBILITY AND COMUNICATION

Data to be cross-checked with government administrative records

1. Because of the CP, have you had to buy any special or rehabilitation equipment?

Yes ☐ No ☐

If YES, please fill in the following table about relevant information on special or rehabilitation equipment that has been purchased by you in the last 12 months:

SPECIAL EQUIPMENT AND AIDS FOR MOBILITY AND COMMUNICATION									
Item name	Purpose of use	Unit price (€)	Quantity purchased	Time of use	Funding Source (Insurance/Gov/Other)	Maintenance/repair cost (€)	Replacement frequency (years)	Additional costs (€)	Access difficulties (Yes/No + comments)

***Examples:** Wheelchairs, crutches, lifts, tablets, communicator, adaptations to special equipment, etc.

5. MODIFICATIONS TO THE HOME AND/OR VEHICLES

2. Because of the patient's illness, have you had to make any modifications to your home or vehicle?

Yes ☐ No ☐

If YES, please fill in the following table with relevant information on modifications that have been made to the household in the last 5 years:

PURCHASE OR MODIFICATION OF HOUSING AND VEHICLES							
Type of modification	Category	Reason	Approximate Price (€)	Out-of-Pocket Cost (€)	Funding Source (Insurance/Gov/Other)	Frequency (No. of times)	Date (Year)

6. CLOTHING, NUTRITION AND PERSONAL CARE: SPECIAL PRODUCTS FOR THE FEEDING AND CARE OF THE PATIENT

3. Have you purchased incontinence products, nutritional products or special clothing and shoes?

Yes ☐ No ☐

If yes, please fill in the following table on the relevant information for the patient in the last 12 months.

CLOTHING, NUTRICIÓN AND PERSONAL CARE								
Item Name	Category	Price per Unit (€)	Price per Pack (€)	Quantity per Purchase	Purchase Frequency (week/month /year)	Total Monthly Cost (€)	Total Annual Cost (€)	Funding Source (Out-of-pocket/Insurance/Support)

***Incontinence:** e.g. nappies. **Nutritional products:** to supplement the patient's diet or because he/she suffers from eating and/or nutritional disorders due to the disease and its comorbidities. **Clothing:** Special clothing and special or adapted shoes.

7. EDUCATION, TRAINING AND INTEGRATION

Data to be cross-checked with government administrative records

3. Does the patient attend a day care center, special school, kindergarten or other?

☐ Yes ☐ No

4. Is this institution public, private or concerted? ____________________

Please fill in the following table if yes for the last 12 months:

EDUCATION, TRAINING AND INTEGRATION							
Institution Name	Type of institution	Start-End Time	She/he has a caretaker at the center	Caregiver's Time (transport + waiting)	Tuition (monthly/semester)	Additional Costs	Transport Costs (EUR)

*Type of institution: Normal, Normal with support, Special center or Special class. Additional cost: Canteen, Excursions or class material, etc.

8. LEISURE, RESPITE CARE AND HOLIDAYS

Please answer this item, if you are the primary caregiver or if you know whether in the last year the patient has incurred expenses for leisure, formal care or holidays.

5. Are there paid a caregiver? Or Do you usually go holiday with the patient?

Yes ☐ No ☐

If the answer is YES, please fill in the following table for the last 12 months:

**Category**	**Type of concept**	**Quantity of time**	**Frequency (weekly/monthly/annual)**	**Cost per unit**	**Total annual cost**	**Funding source**	**Notes**
Formal care							
Leisure							
Holidays							

*Respite care: is considered formal care, as it involves hiring an external care or accessing a specialized service to temporarily relieve the main caregivers

9. DISABILITY ALLOWANCE

a. Has the patient received any disability funding in the last year?

Yes ☐ No ☐

If yes. How much funding did the patient receive in the last 12 months?

A. 400€-800€
B. 850€-1200€
C. 1250€-1600€
D. 1650€-2000€
E. 2050€-4050€
F. More than 5000€
G. You know the exact quantity per year ______________________________

b. Has the main caregiver received any subsidy for care in the last year? Example: CUME

☐ Yes ☐ No

If yes, please answer approximately how much:

A. Lees or up to 1000€
B. 1000€-2000€
C. 2000€-4000€
D. 4000€-8000€
E. 8000€-12000€
F. More than 12000€
G. You know the exact quantity per year ______________________________

10. Other out-of-pocket expenses

a. Do you pay life or health insurance* for your son or daughter?

Yes ☐ No ☐

b. How much do you pay monthly?

A. 0-200 euros ☐
B. 200-300 euros ☐
C. 300-400 euros ☐
D. More than 400 euros ☐

*This question aims to identify the financial burden of private insurance plans, which may be necessary to access treatments not covered by the national health system

Think of any other expenses that have not been mentioned so far. Specify which one(s) and how muchfor each in the last 12 months:

____

____

____

C. Of the out-of-pocket expenses mentioned throughout this questionnaire, which do you consider to have had the greatest impact on your finances?

A. Complementary and Alternative treatments/therapies ☐
B. Special equipment and aids for mobility and communication ☐
C. Modifications to the Home or vehicle ☐
D. Clothing, Nutrition and incontinence products ☐
E. Special education ☐
F. Leisure, Respite Care and Holidays ☐
G. Transportation and fuel ☐
H. Other ______________________________ ☐

C. INDIRECT ECONOMIC BURDEN

11. Loss of Productivity associated with disease care

1. **How long have you been providing care or assistance to the patient?**

   A. Less than one year ☐
   B. *1 to 2 years* ☐
   C. *From 2 to 4 years old* ☐
   D. *From 4 to 8 years old* ☐
   E. *More than 8 years* ☐
   F. *Don't know / No answer* ☐

2. **Could you tell me what are the main tasks in which you help or care for this person?**

   A. Eating, drinking, feeding ☐
   B. Dressing, undressing, fastening shoes, grooming, dressing up ☐
   C. Bathing, showering ☐
   D. Going to the toilet, changing nappies, etc. ☐
   E. Changing posture, moving or holding the body in a certain position ☐
   F. Walking or moving around the house ☐
   G. Going outdoors, going up or down stairs, walking, using public transport ☐
   H. Taking medication, attending medical appointments, therapies etc. ☐
   I. Doing rehabilitation at home ☐
   J. Shopping, preparing special meals, etc. ☐
   K. Continuous monitoring ☐
   L. Support for learning and communication ☐
   M. Other tasks: ☐

Specify which ones: ....

3. **How many days a week, on average, do you spend part of your time helping or caring for this person**?

   A. 1 day *(per week)* ☐
   B. 2 days *(per week)* ☐
   C. 3 days *(per week)* ☐
   D. 4 days *(per week)* ☐
   E. 5 days *(per week)* ☐
   F. 6 days *(per week)* ☐
   G. 7 days a week ☐
   H. *Don't know / No answer* ☐

4. How many hours, on average, do you spend caring for this person each day that you provide assistance?

A. Less than one hour ☐
B. 2 hours ☐
C. 4 hours ☐
D. 8 hours ☐
E. 12 hours ☐
F. 16 hours ☐

Diary: Informal care monitoring module to calculate productivity loss

[Instruction: Please complete this diary every day for 4 consecutive weeks. Do not exceed 16 hours per day.]

In the following, you will be presented with a diary containing in general the different activities that you usually do with the disabled child. The activities have been selected on the basis of the survey on disability, personal autonomy and dependency situations. This diary is divided by weeks, of which there are 4, and within each weekby days, of which there are 7 for each week. As this is a diary you are asked to try to fill it in every day for 4 weeks. Please note that you cannot exceed 16 hours per day. That is, the sum of each of the activities must be equal to or less than 16 hours, it cannot be more. This is because, even if you spend the whole day caring for your child, there is a margin of time you have between tasks to spend on yourself and your basic needs.

1. **Could you tell me how many hours a day you spend on the tasks in which you assist or care for the patient?**

MONTHLY CARE ACTIVITIES DIARY

HOURS PER DAY THAT YOU SPEND ASSISTING OR CARING FOR YOUR FAMILY MEMBER WITH CP IN THE FOLLOWING ACTIVITIES

Days	1. Eating, drinking, feeding	2. Dressing, undressing, fastening shoes	3. Bathing, showering	4. Go to service, change nappies	5. Changing posture, moving or keeping the body in a certain position	6.Walking or moving around the house	7. Going outside going up or down stairs, taking walks, using transport public	8. Taking medication, attending medical consultations or therapies	9. Doing rehabilitation at home	10. Preparing special meals	11. Continuous monitoring	12. Support for learning and communication	Other chores in relation with care	Total hours per day
	No. Minutes /Hours	No. Minutes /Hours	No. Minutes /Hours	No. Minutes /Hours	No. Minutes /Hours	No. Minutes /Hours	No. Minutes /Hours	No. Minutes /Hours	No. Minutes /Hours	No. Minutes /Hours	No. Minutes /Hours	No. Minutes /Hours	No. Minutes /Hours	
**Week 1**														
1														
2														
3														
4														
5														
6														
7														
**Week 2**														
8														
9														
10														
11														
12														
13														
14														
**Week 3**														
15														
16														
17														
18														
19														
20														
21														
**Week 4**														
22														
23														
24														
25														
26														
27														
28														
29														
30														

*As a conservative criterion, and to avoid co-production bias, we censor care time to a maximum of 16 h per day per caregiver (112 h per week) when the reported time exceeds this figure. The approach used to assess hours of care will be the proxy good method, which values time as an outcome.

QUESTIONS 2-12: IMPACT OF CARE AND FINANCIAL SITUATION: Caregiver wellbeing

2. **What do you consider to have been the main activity that has cost you the most time in caringfor the patient (choose the one that has had the greatest impact on your life)?**

3. **Concerning matters relating to their health or general condition and related to the help or care theyprovide to that person:**

[Scale clarification: 1 = Lowest possible extent, 10 = Highest possible extent]

a. Do you consider that your physical health has deteriorated in the last 12 months?

Yes☐ No☐ Don't know / No answer ☐

1 2 3 4 5 6 7 8 9 10

____

(Enter a number from 1-10)

b. Do you consider that your mental health has deteriorated in the last 12 months?

Yes☐ No☐ Don't know / No answer ☐

1 2 3 4 5 6 7 8 9 10

____

(Enter a number from 1-10)

c. Do you consider that your social life has deteriorated in the last 12 months?

Yes☐ No☐ Don't know / No answer ☐

1 2 3 4 5 6 7 8 9 10

____

(Enter a number from 1-10)

d. Have you had to undergo treatment to cope with the physical situation in the last 12 months?
(Antidepressants, nerves, anxiety, anxiety, distress,)

Yes☐ No☐ Don't know / No answer☐

Type oftreatment:

Medical ☐
Psychological ☐
Specify which ones ___________________

Where the service comes from:

1) Public ☐
2) Private ☐

e. Approximately how much did this treatment cost you?

A. 100 -200 euros ☐
B. 200- 400 euros ☐
C. 400-800 euros ☐
D. More than 800 euros ☐

4. After covering all your monthly expenses, do you have any money left over to save?
- ☐ Never
- ☐ Rarely
- ☐ Sometimes
- ☐ Always

1 2 3 4 5 6 7 8 9 10

____

(Enter a number from 1-10)

5. What do you feel is the level of your financial stress today?
- ☐ Overwhelming stress
- ☐ High stress
- ☐ Low stress
- ☐ No stress at all

1 2 3 4 5 6 7 8 9 10

____

(Enter a number from 1-10)

6. How satisfied are you with your present financial situation?
- ☐ Dissatisfied
- ☐ Satisfied

1 2 3 4 5 6 7 8 9 10

____

(Enter a number from 1-10)

7. How do you feel about your current financial situation?
- ☐ Feel overwhelmed
- ☐ Sometimes feel worried
- ☐ Rarely worry
- ☐ Never worry

1 2 3 4 5 6 7 8 9 10

____

(Enter a number from 1-10)

8. How often do you worry about being able to meet normal monthly living expenses?

- ☐ Worry all the time
- ☐ Sometimes worry
- ☐ Rarely worry
- ☐ Never worry

1 2 3 4 5 6 7 8 9 10

____

(Enter a number from 1-10)

9. How confident are you that you could find the money to pay for a financial emergency that costs about €1000?

- ☐ No confidence
- ☐ Little confidence
- ☐ Some confidence
- ☐ High confidence

1 2 3 4 5 6 7 8 9 10

____

(Enter a number from 1-10)

10. How often does this happen to you? You want to go out to eat, go to a movie or do something else and don't go because you can't afford it?

- ☐ All the time
- ☐ Sometimes
- ☐ Rarely
- ☐ Never

1 2 3 4 5 6 7 8 9 10

____

(Enter a number from 1-10)

11. How frequently do you find yourself just getting by financially and living day to day?

- ☐ All the time
- ☐ Sometimes
- ☐ Rarely
- ☐ Never

1 2 3 4 5 6 7 8 9 10

____

(Enter a number from 1-10)

12. How stressed do you feel about your personal finances in general?

- ☐ Overwhelming stress
- ☐ High stress
- ☐ Low stress
- ☐ No stress at all

1 2 3 4 5 6 7 8 9 10

____

(Enter a number from 1-10)

THANK YOU VERY MUCH FOR YOUR PARTICIPATION
