# Supplementary material for: The economic and social burden of pediatric cerebral palsy in Spain: a cost-of-illness study
Source: Front Public Health. 2025 Jul 23;13:1589114. doi: 10.3389/fpubh.2025.1589114 (PMC12325214; doi:10.3389/fpubh.2025.1589114)
Supplement: Supplementary file 2 [file Supplementary_file_1.docx]

**Supplementary Material: The Economic and Social Burden of Pediatric Cerebral Palsy in Spain: A Cost-of-Illness Study**

**
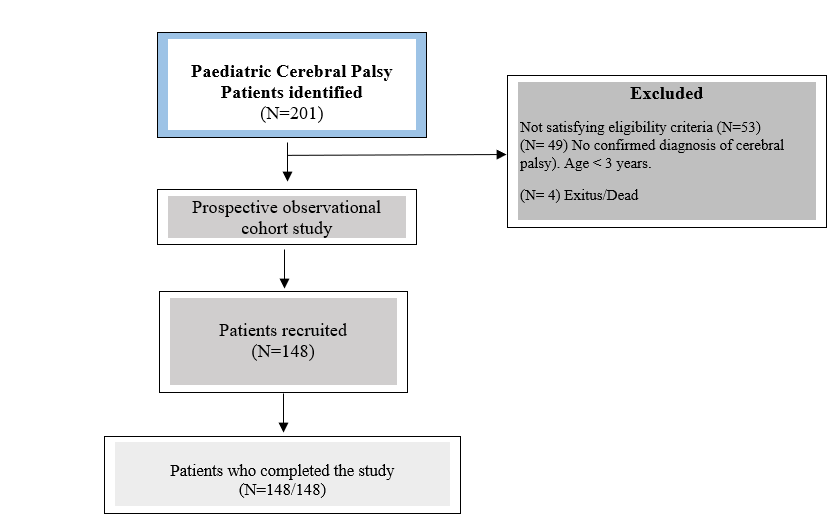
Figure S1.** Flowchart of patient selection and inclusion process in the study population with pediatric cerebral palsy.
